# Evolution and consequences of individual responses during the COVID-19 outbreak

**DOI:** 10.1371/journal.pone.0273964

**Published:** 2022-09-01

**Authors:** Wasim Abbas, Masud M. A., Anna Park, Sajida Parveen, Sangil Kim

**Affiliations:** 1 Department of Mathematics, Pusan National University, Busan, Korea; 2 Natural Product Informatics Research Center, Korea Institute of Science and Technology, Seoul, Gangneung, South Korea; 3 Institute of Mathematical Sciences, Pusan National University, Busan, Korea; Nanyang Technological University, SINGAPORE

## Abstract

In a long-lasting major disease outbreak such as that of COVID-19, the challenge for public health authorities is to keep people motivated and keen on following safety guidelines. In this study, a compartmental model with a heterogeneous transmission rate (based on awareness) is utilized to hypothesize about the public adoption of preventive guidelines. Three subsequent outbreaks in South Korea, Pakistan, and Japan were analyzed as case studies. The transmission, behavior change, and behavioral change ease rates of the disease were measured in these countries. The parameters were estimated using the maximum likelihood method with an additional identifiability analysis performed to determine the uniqueness of the estimated parameters for quantitatively comparing them during the first three waves of COVID-19. The mathematical analysis and simulation results show that individual responses had a significant effect on the outbreak. Individuals declining to follow the public health guidelines in Korea and Japan between the second and third waves contributed to making the third peak the highest of the three peaks. In Pakistan, however, individual responses to following public health guidelines were maintained between the second and third waves, resulting in the third peak being lower than the first, rather than being associated with the highest transmission rate. Thus, maintaining a high level of awareness is critical for containing the spread. Improvised public health campaigns are recommended to sustain individual attention and maintain a high level of awareness.

## Introduction

The coronavirus disease (COVID-19) is an infectious disease caused by the SARS-CoV-2 virus. Most people infected with the virus experience mild-to-moderate respiratory symptoms and recover without special treatment. However, some patients can become seriously ill and may require medical attention. Older people and those with underlying medical conditions such as cardiovascular diseases, diabetes, chronic respiratory illnesses, or cancer, are more likely to develop serious illnesses following COVID-19 infection. Anyone can get infected with COVID-19 and serious consequent health issues, including death, can never be ruled out with certainty. Chinese authorities have identified this outbreak as a new coronavirus, differing completely from other coronaviruses previously encountered among humans [1]. The novel coronavirus disease (COVID-19) has become a global threat to public health and the economy. On January 30, 2020, the World Health Organization (WHO) announced a public health emergency of international concern (PHEIC) [2].

Non-pharmaceutical interventions for reducing the probability of infectious contact are the primary strategies for stopping outbreaks when pharmaceutical tools, such as vaccines and medicines are scarce [3]. Due to the short-term existence of both natural and vaccine-induced immunity against SARS-CoV-2 [4], non-pharmaceutical interventions hold a significant role in containing the outbreak [5]. Although COVID-19 is a flu-like illness, its high transmissibility and pandemic nature significantly affect daily human life and communication in this fast-moving world [6]. Individual disease experiences and beliefs govern the behavioral factors associated with disease outbreaks [7]. Due to the long-term persistence and subsequent outbreaks of several strains of COVID-19, maintaining social distance and associated preventive measures has become challenging [8].

Human behavior evolves in response to epidemic outbreaks [9]. Though fear of disease increases at the beginning of an outbreak, frustration or boredom associated with stringent preventive measures relaxes it with time [10]. A social network may also shape the awareness and response to preventive measures [11, 12]. Societal reactions affect the spread of epidemics, as discussed in [13, 14]. Further, understanding the incentives for plausible infectious contact is critical for forming and implementing effective social distancing policies [15]. For instance, during the 1918 influenza outbreak in the USA, individuals realized the higher cost associated with contact as a consequence of the high mortality rate and proactively adopted social distancing [16]. Public health policies may exacerbate the situation and the disease may become more prevalent when these measures nominate susceptible individuals for lower incentives [17].

Conversely, behavioral change may shape the outbreak [18]. In [19], it was shown that behavioral changes may modulate the uncertainty of the peak under both deterministic and stochastic model settings. Understanding spontaneous behavioral changes can be helpful for health policymakers in planning public health control strategies and estimating the burden on healthcare centers over time [20]. An increased sense of safety because of vaccination may cause aware individuals to loosen the preventive measures [21]. Overcoming fear was identified as a cause of multiple epidemic waves [22] while avoiding unnecessary fear sentiment is recommended [13]. As a result, there is a lack of qualitative and quantitative understanding of fear-induced behavioral changes associated with implementing preventive measures and their implications for disease spread.

An effort toward modeling fear-induced behavioral change showed that publicly available information on infection spread results in several waves by stimulating mass acceptance of preventive measures [23]. Applying a similar modeling assumption to a meta-populational framework, Massaro et al. showed that intervention measures might also delay the resilience of societal factors [24]. A cross-country comparison of the COVID-19 outbreak showed that behavioral changes were adopted quicker in South Korea than in Italy, which resulted in more successful containment in the former country than in the latter during the first wave [25]. However, in the Daegu/Gyeongbuk area of South Korea, the behavioral change rate was low compared to the rest of the country, which resulted in a significantly larger outbreak in the respective area [26]. In another preprint [27], a behavior-disease model incorporated with the government-imposed public health measures was fitted to the first wave of the COVID-19 outbreak in the city of Metro Manila, Philippines. This study showed that the behavioral change rate decreased towards the end of the wave. However, several outbreaks of COVID-19 have been observed throughout the world so far. So, it is interesting to investigate the evolution of behavioral change over subsequent outbreaks and its relevance to it.

We extend the behavior-disease modeling approach proposed in [28]. The model was fitted to each of the first three COVID-19 waves that occurred in South Korea, Pakistan, and Japan. It is important to accurately associate unknown parameters with the values that relate the model to the data. For a comprehensive analysis of the modeling results, it is also necessary to determine the reliability of the parameter estimates. The identifiability analysis guarantees that the estimated parameters are uniquely determined. If the parameters are identifiable, the parameter estimation is independent of the initial guess of the unknown parameters. Ensuring the identifiability of the parameters, a model was fitted to explore the change in individual responses to public health guidelines, demonstrating that the tendency to adopt preventive measures inspired by public health initiatives has decreased over time in Korea and Japan but is maintained in Pakistan.

## Materials and methods

### Mathematical modeling

**Fig 1:**
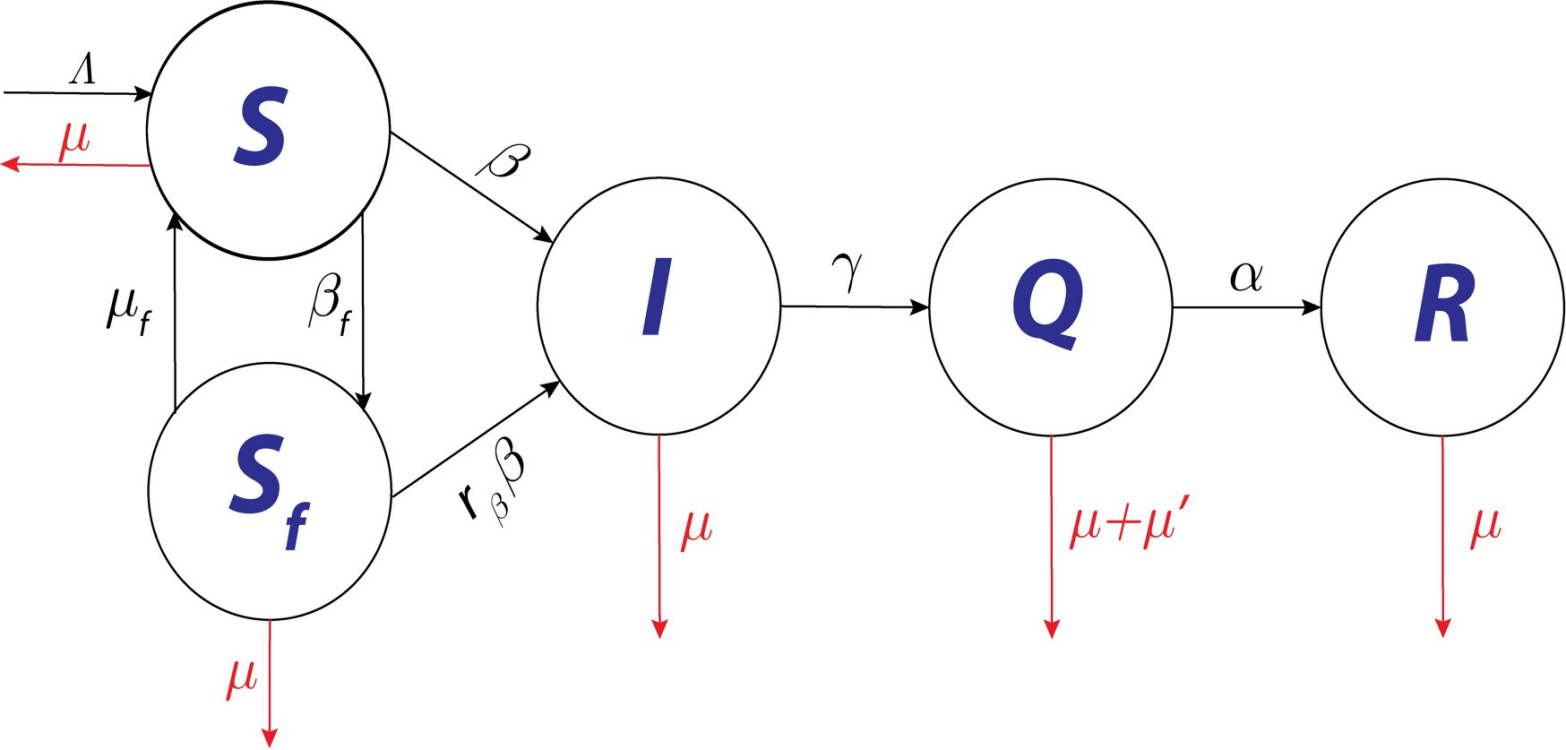
Schematic diagram of the proposed mathematical model.

As a modification to the model proposed in [28], heterogeneity in transmission is assumed based on the level of awareness of individuals. The proposed model consists of five classes, where the total population *N*(*t*) was partitioned into classes *S*(*t*), *S*_*f*_(*t*), *I*(*t*), *Q*(*t*), and *R*(*t*). These classes denote susceptible, behavior-changed susceptible, infectious, active (quarantined or hospitalized), and recovered individuals, respectively. A schematic representation of this model is shown in Fig 1.

The system of equations associated with the aforementioned diagram can be expressed as:

$$\frac{dS}{dt}=\Lambda -\frac{\beta SI}{N}-\beta_{f}S(1-e^{-\delta Q})+\mu_{f}S_{f}(\frac{S+R}{N})-\mu S$$ (1)

$$\frac{dS_{f}}{dt}=-\frac{r_{\beta }\beta S_{f}I}{N}+\beta_{f}S(1-e^{-\delta Q})-\mu_{f}S_{f}(\frac{S+R}{N})-\mu S_{f}$$ (2)

$$\frac{dI}{dt}=\frac{\beta SI}{N}+\frac{r_{\beta }\beta S_{f}I}{N}-(\gamma +\mu )I$$ (3)

$$\frac{dQ}{dt}=\gamma I-(\alpha +\mu +\mu^{\prime })Q$$ (4)

$$\frac{dR}{dt}=\alpha Q-\mu R$$ (5)

In the susceptible class *S*, individuals become infected with the transmission rate *β* and move to the infectious class *I*. When the number of infected cases increases in society, fear of the disease prevails in susceptible individuals. With the behavior change rate (transmission rate of fear) *β*_*f*_, susceptible individuals move to the behavior-changed susceptible class *S*_*f*_. Because of this behavioral change, the transmission rate in *S*_*f*_ is lowered by a fraction *r*_*β*_ (0≤*r*_*β*_≤1). Therefore, individuals from *S*_*f*_ move to infectious class *I* with a lower transmission rate, *r*_*β*_*β* than those from *S*. Strictly following social distancing, mask-wearing, and avoiding social gatherings are considered as behavioral changes in the proposed model. After confirmation, infectious individuals move to quarantine class *Q* with a confirmation rate *γ* and confirmed infected individuals *Q*, move to the recovered class with a rate of recovery *α*. Individuals in the model are recruited to susceptible class *S* with a constant migration rate Λ, and they are removed from all classes upon natural death rate *μ*. In quarantined classes, individuals are also removed with disease-related death rate *μ*′. Individuals in *S*_*f*_ influenced by the susceptible and recovered individuals, become hesitant to strictly follow the preventive measures and revert to the susceptible class at a behavioral change ease rate of *μ*_*f*_.

### Mathematical analysis

The proposed mathematical model was analyzed to gain an understanding of the dynamics of models (1)-(5) from an epidemiological perspective.

The model exhibits three equilibria: trivial, (0,0,0,0,0) disease-free (*S*_0_,0,0,0,0), and endemic equilibrium $\left(S^{*},S_{f}^{*},I^{*},Q^{*},R^{*}\right)$. However, $\frac{\beta SI}{N}$ and $\frac{r_{\beta }\beta S_{f}I}{N}$ are not differentiable at the trivial equilibrium. Therefore, stability analysis at this equilibrium was not feasible using standard linearization methods. The existence and uniqueness of the remaining equilibria are described in the following propositions:

**Proposition 1:** The system of Eqs (1)–(5) has a unique disease-free equilibrium *E*_0_, defined as *E*_0_ = (*S*_0_,0,0,0,0). Here, $S_{0}=\frac{\Lambda }{\mu }$.

**Proof.** For the disease-free equilibrium, the left-hand side of the system of equations was set as equal to 0, and it was assumed that there was no disease in the beginning.

Therefore, *I* = 0, implies *Q* = 0 and *R* = 0. From the first and second equations of the system, it is determined that:

$$\Lambda +\frac{\mu_{f}S_{f}S}{N}-\mu S=0$$ (6)

$$-\frac{\mu_{f}S_{f}S}{N}-\mu S_{f}=0$$ (7)

Eq (7) implies that $-\mu_{f}\frac{S}{N}-\mu =0$ or *S*_*f*_ = 0. If *S*_*f*_≠0, then

$$\mu_{f}\frac{S}{N}=-\mu ⟹\frac{S}{N}=-\frac{\mu }{\mu_{f}}.$$

This contradicts the fact that *S*, *N*≥0; therefore, *S*_*f*_ will always be 0 in a disease-free equilibrium. This is biologically meaningful, as it is natural to assume that all susceptible individuals are fear-free in the beginning. In addition, after time *t*, if the disease wanes, the fear of the disease subsides in society and behavioral changes return to normal in the susceptible population.

Letting *S*_*f*_ = 0, we obtain Eq (6) as $S=\frac{\Lambda }{\mu }$. Thus, there is a unique disease-free equilibrium $E_{0}=(\frac{\Lambda }{\mu },0,0,0,0)$ in the model.∎

**Proposition 2:** The system of Eqs (1)–(5) has a unique endemic equilibrium, E*, defined as $E^{*}=(S^{*},S_{f}^{*},I^{*},Q^{*},R^{*}).$ Here,

$$S^{*}=\frac{(1-r_{\beta }R_{0})N^{*}}{(1-r_{\beta })R_{0}}-\frac{r_{\beta }(\Lambda -\mu N^{*})(1-R_{1})}{\mu (1-r_{\beta })},$$

$$S_{f}^{*}=\frac{\left(\Lambda -\mu N^{*})(1-R_{1}\right)}{\mu (1-r_{\beta })}+\frac{(R_{0}-1)N^{*}}{(1-r_{\beta })R_{0}},$$

$$I^{*}=\frac{\left(\mu +\alpha +\mu^{\prime })(\Lambda -\mu N^{*}\right)}{\mu^{\prime }\gamma },Q^{*}=\frac{\left(\Lambda -\mu N^{*}\right)}{\mu^{\prime }},$$

and $R^{*}=\frac{\alpha (\Lambda -\mu N^{*})}{\mu \mu^{\prime }}$. While $R_{0}=\frac{\beta }{\gamma +\mu },R_{1}=\frac{\left(\mu +\alpha +\mu^{\prime })(\mu +\gamma \right)}{\mu^{\prime }\gamma }$ and

$$N^{*}=S^{*}+S_{f}^{*}+I^{*}+Q^{*}+R^{*}.$$

**Proof.** For the endemic equilibrium, it is assumed that *I*≠0. Using this assumption, the uniqueness of the endemic equilibrium can be easily proved.∎

### Stability analysis

The basic reproduction number, *R*_0_, plays a crucial role in the analysis of single-population outbreaks, which are the total expected secondary cases that the primary infected individual generates. To determine *R*_0_, the approach defined in [29] is used. Thus, the next generation matrix, *J*, in our model is

$$J=(\begin{array}{cc} \frac{\beta }{\mu +\gamma } & 0\\ 0 & 0 \end{array}).$$

The eigenvalues of *J* are $\frac{\beta }{\mu +\gamma }$ and 0, which implies that the spectral radius of *J* is $\frac{\beta }{\mu +\gamma }$.

Hence,

$$R_{0}=\frac{\beta }{\mu +\gamma }.$$ (8)

**Lemma 1:** Disease-free equilibrium *E*_0_ is locally asymptotically stable in domain $R_{+}^{5}$ if and only if the basic reproduction number *R*_0_, is less than 1.

**Proof.** If there is no infectious individual, then Proposition 1 ensures the uniqueness of disease-free equilibrium *E*_0_. We show that this unique disease-free equilibrium *E*_0_, is locally asymptotically stable. The Jacobian matrix of the system of Eqs (1)–(5) at *E*_0_ is

$$B=\left(\begin{array}{cc} B_{1} & B_{2}\\ B_{3} & B_{4} \end{array}\right).$$

$$Here,\;B_{1}=\left(\begin{array}{cc} -\mu & \mu_{f}\\ 0 & -(\mu +\mu_{f}) \end{array}\right),B_{2}=\left(\begin{array}{ccc} -\beta & -\delta \beta_{f}S_{0} & 0\\ 0 & \delta \beta_{f}S_{0} & 0 \end{array}\right),$$

$$B_{3}=O_{3\times 2},\;and\;B_{4}=(\begin{array}{ccc} \beta -(\mu +\gamma ) & 0 & 0\\ \gamma & -(\alpha +\mu +\mu^{\prime }) & 0\\ 0 & \alpha & -\mu \end{array}).$$

The eigenvalues of $B_{1}$ are −*μ* and −(*μ*+*μ*_*f*_) and the eigenvalues of $B_{4}$ are

−*μ*, −(*α*+*μ*+*μ*′) and *β*−(*μ*+*γ*). Therefore, if $R_{0}=\frac{\beta }{\mu +\gamma }<1$, all eigenvalues of B have negative real parts. Therefore, according to the Routh–Hurwitz stability criterion, *E*_0_ is locally asymptotically stable in $R_{+}^{5}$.

**Lemma 2:** Endemic equilibrium E* is locally asymptotically stable in domain $R_{+}^{5}$ if and only if the basic reproduction number *R*_0_ is greater than 1.

**Proof.** We prove this result by using the center manifold theorem. Let *ϕ* be the bifurcation parameter. If *y*_1_ = *S*, *y*_2_ = *S*_*f*_, *y*_3_ = *I*, *y*_4_ = *Q*, *y*_5_ = *R* and *y* = *y*_1_+*y*_2_+*y*_3_+*y*_4_+*y*_5_, the system of equations can be expressed as follows:

$$\frac{dy_{1}}{dt}=\Lambda -\frac{\beta y_{1}y_{3}}{y}-\beta_{f}y_{1}(1-e^{-\delta y_{4}})+\mu_{f}y_{2}(\frac{y_{1}+y_{5}}{y})-\mu y_{1}=g_{1}$$ (9)

$$\frac{dy_{2}}{dt}=-\frac{r_{\beta }\beta y_{2}y_{3}}{y}+\beta_{f}y_{1}(1-e^{-\delta y_{4}})-\mu_{f}y_{2}(\frac{y_{1}+y_{5}}{y})-\mu y_{2}=g_{2}$$ (10)

$$\frac{dy_{3}}{dt}=\frac{\beta y_{1}y_{3}}{y}+\frac{r_{\beta }\beta y_{2}y_{3}}{y}-(\gamma +\mu )y_{3}=g_{3}$$ (11)

$$\frac{dy_{4}}{dt}=\gamma y_{3}-(\alpha +\mu +\mu^{\prime })y_{4}=g_{4}$$ (12)

$$\frac{dy_{5}}{dt}=\alpha y_{4}-\mu y_{5}=g_{5}$$ (13)

The linearization matrix, $D_{y}g$, of the aforementioned system of equations around *E*_0_, when *ϕ* = ϕ*, is given by,

$$D_{y}g=\left(\begin{array}{ccccc} -\mu & \mu_{f} & -\phi^{*} & -\frac{\delta \beta_{f}\Lambda }{\mu } & 0\\ 0 & -(\mu +\mu_{f}) & 0 & \frac{\delta \beta_{f}\Lambda }{\mu } & 0\\ 0 & 0 & \phi^{*}-(\mu +\gamma ) & 0 & 0\\ 0 & 0 & \gamma & -(\alpha +\mu +\mu^{\prime }) & 0\\ 0 & 0 & 0 & \alpha & -\mu \end{array}\right).$$

It is observed that 0 is a simple eigenvalue of this matrix. Therefore, let *H* = [*h*_1_, *h*_2_, *h*_3_, *h*_4_, *h*_5_]^*t*^ be the right eigenvector of $D_{y}g$ such that,

$$\left(\begin{array}{ccccc} -\mu & \mu_{f} & -\phi^{*} & -\frac{\delta \beta_{f}\Lambda }{\mu } & 0\\ 0 & -(\mu +\mu_{f}) & 0 & \frac{\delta \beta_{f}\Lambda }{\mu } & 0\\ 0 & 0 & \phi^{*}-(\mu +\gamma ) & 0 & 0\\ 0 & 0 & \gamma & -(\alpha +\mu +\mu^{\prime }) & 0\\ 0 & 0 & 0 & \alpha & -\mu \end{array}\right)\left(\begin{array}{c} h_{1}\\ h_{2}\\ h_{3}\\ h_{4}\\ h_{5} \end{array}\right)=(\begin{array}{c} 0\\ 0\\ 0\\ 0\\ 0 \end{array}).$$

Thus, the right eigenvector, *H*, is given by,

$$H={\left[-\frac{1}{\mu }(\phi^{*}+\frac{\delta \beta_{f}\Lambda \gamma }{\left(\mu +\mu_{f})(\alpha +\mu +\mu^{\prime }\right)}),\frac{\delta \beta_{f}\Lambda \gamma }{\mu (\mu +\mu_{f})(\alpha +\mu +\mu^{\prime })},1,\frac{\gamma }{\alpha +\mu +\mu^{\prime }},\frac{\alpha \gamma }{\mu (\alpha +\mu +\mu^{\prime })}\right]}^{t}.$$

Similarly, the left eigenvector of matrix $D_{y}g$ is

$$C={\left[0,0,1,0,0\right]}^{t}.$$

Therefore, coefficients *a* and *b* are defined as

$$a=\sum_{k,i,j=1}^{5}c_{k}h_{i}h_{j}\frac{\partial^{2}g_{k}}{\partial y_{i}\partial y_{j}}(E_{0}),\;\mathrm{and}$$

$$b=\sum_{k,i=1}^{5}c_{k}h_{i}\frac{\partial^{2}g_{k}}{\partial y_{i}\partial \phi }.$$

By substituting the values,

$$a=\frac{2\delta \beta_{f}\Lambda \gamma }{\mu (\mu +\mu_{f})(\alpha +\mu +\mu^{\prime })}\left(\frac{\mu \phi }{\Lambda }(r_{\beta }-1)\right)-\frac{2\mu \phi }{\Lambda }(1+\frac{\gamma }{\alpha +\mu +\mu^{\prime }}+\frac{\alpha \gamma }{\mu (\alpha +\mu +\mu^{\prime })}),$$

where *b* = 1, when *ϕ*>0, *a*<0, and *b*>0. Therefore, based on Theorem 4.1 in [30], the endemic equilibrium E* is locally asymptotically stable if and only if *ϕ*>0. This implies that E* is locally asymptotically stable if and only if *R*_0_>1. ∎

### Parameterization

The descriptions and values of the parameters used in the system of equations are presented in Tables 1 and 2. These parameters are used to solve the system of Eqs (1)–(5). In Table 1, the parameters are adopted from references or estimated from the data.

**Table 1:** Parameters sourced from relevant sources or estimated from available data. Here, D.L. indicates dimension lessness.

Symbol	Description		Value	Unit	Reference
Λ	Immigration rate	Korea	855	day^−1^	Estimated
		Pakistan	16953	day^−1^	Estimated
		Japan	2413	day^−1^	Estimated
*μ*	Natural mortality rate	Korea	$\frac{1}{83}\times \frac{1}{365}$	day^−1^	Estimated
		Pakistan	$\frac{1}{67}\times \frac{1}{365}$	day^−1^	Estimated
		Japan	$\frac{1}{85}\times \frac{1}{365}$	day^−1^	Estimated
*r*_*β*_	Transmission reduction ratio of behaviorally changed individuals		*0*.*02*	D.L.	[28]
*γ*	Isolation rate		$\frac{1}{4}$	day^−1^	[28]
*μ*′	Disease induced death rate	Korea	*0*.*0221*	day^−1^	[28]
		Pakistan	0.0202	day^−1^	Estimated
		Japan	0.0238	day^−1^	Estimated
*α*	Recovery rate		$\frac{1}{14}$	day^−1^	[28]
1/*δ*	Characteristic number of confirmed individuals reported by news		1000	day^−1^	[28]

**Table 2:** Parameters estimated using the maximum likelihood method. Here, C.I. stands for the confidence interval.

Symbol	Description		Values		
			1^st^ Wave	2^nd^ Wave	3^rd^ Wave
*β*	Transmission rate	Korea C.I.	0.3619 (0.3598, 0.3636)	0.6445 (0.6374, 0.6516)	0.9820 (0.9749, 0.9892)
		Pakistan C.I.	0.3966 (0.3960,0.3972)	1.1177 (1.1023, 1.1338)	1.7896 (1.7792, 1.8002)
		Japan C.I.	0.5310 (0.5242, 0.5380)	0.8318 (0.8291, 0.8345)	1.3023 (1.2759, 1.3315)
*β*_*f*_	Transmission rate of the fear of the disease	Korea C.I.	0.0517 (0.0506, 0.0527)	0.3245 (0.3149, 0.3346)	0.0690 (0.0640, 0.0742)
		Pakistan C.I.	0.0098 (0.0097, 0.0099)	0.1733 (0.1686, 0.1781)	0.1766 (0.1727, 0.1805)
		Japan C.I.	0.0642 (0.0601, 0.0686)	0.2174 (0.2123, 0.2226)	0.1722 (0.1500, 0.1958)
*μ*_*f*_	Behavioral change ease rate	Korea C.I.	0.1131 (0.1115, 0.1147)	0.4426 (0.4307, 0.4551)	0.0813 (0.0749, 0.0879)
		Pakistan C.I.	0.0139 (0.0136, 0.0141)	0.2134 (0.2084, 0.2185)	0.1900 (0.1859, 0.1941)
		Japan C.I.	0.0802 (0.0739, 0.0869)	0.2935 (0.2668, 0.3003)	0.2070 (0.1806, 0.2350)

According to World Bank, the crude birth rate per year in the Republic of Korea is $\frac{6}{1000}$ (see [31]) and the total population is approximately 52 million. Therefore, the number of new births per year is 312,000 and the immigration rate per day is $\frac{312000}{365}\approx 855$. Similarly, the migration rate for Pakistan and Japan is 16953 and 2413, respectively.

The average life expectancy in South Korea is approximately 83 years [32]; therefore, the natural death rate in Korea is $\frac{1}{83}\times \frac{1}{365}$ per day. The average life expectancy in Pakistan is 67 years [32]. Therefore, the natural death rate in Pakistan is $\frac{1}{67}\times \frac{1}{365}$. Similarly, the natural death rate in Japan is $\frac{1}{85}\times \frac{1}{365}$ [32].

We estimate Pakistan’s disease-related death rate on average is, $\left(\frac{\mathrm{Deaths}\;\mathrm{due}\;\mathrm{to}\;\mathrm{disease}}{\mathrm{Confirmed}\;\mathrm{cases}}\right)=0.0202$ [33]. Furthermore, the disease-related death rate in Japan is 0.0238.

In Table 2, the remaining parameters are estimated by using the maximum likelihood method. In particular, three parameters are estimated for each of the three waves. The period of three waves in Korea was divided into: first wave (February 16, 2020 to August 11, 2020), second wave (August 11, 2020 to November 11, 2020), and third wave (November 11, 2020, to February 14, 2021) based on [33, 34] (please refer to S1 Fig and S1 Table in S1 File).

For Pakistan, the timeline of the three peaks was defined as: first wave (March 17, 2020, to October 13, 2020), second wave (October 13, 2020, to March 7, 2021), and third wave (March 7, 2021, to June 20, 2021) based on [33] (please refer to S2 Fig and S2 Table in S1 File).

The timeline of the three peaks for Japan was described as follows: first wave (March 10, 2020 to June 28, 2020), second wave (June 28, 2020 to October 21, 2020), and third wave (October 21, 2020 to February 14, 2021) based on [33] (please refer to S3 Fig and S3 Table in S1 File).

For all three waves, the initial value of the infectious population is estimated using the maximum-likelihood method. Fear at the start of the disease is negligible; therefore, *S*_*f*_(0) = 0 in the first wave. *S*_*f*_ is also modified to *N*−*S*−*I*−*Q*−*R* in our system of equations.

It is observed that with an increase in the number of unknown parameters, identifiability issues are raised. To address these issues, a code in MATLAB is executed for the first wave up to the initial value of the second wave and considering *S*(178) = 34.271×10^6^, *S*(211) = 99.275×10^6^ and *S*(111) = 55.487×10^6^ as the initial values for the second wave of Korea, Pakistan, and Japan, respectively.

Similarly, as in the second wave, we estimated *S*(0) = 18.691×10^6^, *S*(0) = *43.773*×10^6^ and *S*(0) = *33.291*×10^6^ for the third wave of the respective countries.

### Fitting the temporal data

In the formulation, *β* is the transmission rate; *β*_*f*_ is the behavior change rate; *μ*_*f*_ is the behavioral change ease rate; and *I*(0) is the initial value of the infectious population. As data on the infectious population were unavailable, estimating these unknowns directly from the data was impossible. Thus, the parameters *β*, *β*_*f*_, *μ*_*f*_, and *I*(0) were estimated using the maximum likelihood method. Time-series data were employed for active infected cases ([33]). The details of the time-series data are provided for those countries in S1–S3 Tables in S1 File.

Let $X=(x_{1},x_{2},x_{3},x_{4},x_{4})=(S,S_{f},I,Q,R)\in R_{+}^{5}$ be the vector of state variables; F is the right side of the system ((1)-(5)); ℙ = (*β*, *β*_*f*_, *μ*_*f*_, *I*(0)) is the vector of the unknowns to be estimated; *Q*(*t*, ℙ) be the vector of observables, and *Q*^0^(*t*, ℙ) is the observed data at *t*. Here, *t* is the time interval for the first wave (the assumptions were similar for the second and third waves). It is assumed that all *Q*^0^(*t*, ℙ) are independent and drawn from the Poisson distribution with a mean equal to *Q*(*t*, ℙ); then, the Poisson maximum likelihood function is

$$L(Q(t,P)|Q^{0}(t,P))=\prod_{i=1}^{n}\frac{Q{\left(t_{i}\right)}^{Q_{i}^{0}}\cdot e^{-Q(t_{i})}}{Q_{i}^{0}!}.$$

Therefore, the negative log-likelihood function (NLF) reduces to

$$\mathrm{NLF}=-\ln\left(\prod_{i=1}^{n}\frac{Q{\left(t_{i}\right)}^{Q_{i}^{0}}\cdot e^{-Q(t_{i})}}{Q_{i}^{0}!}\right)$$

$$=-\sum_{i=1}^{n}Q_{i}^{0}\ln Q(t_{i})+\sum_{i=1}^{n}Q(t_{i})+\sum_{i=1}^{n}\ln Q_{i}^{0}!.$$

Because the last term in the aforementioned equation is constant, it remains unchanged as the parametric values vary. Therefore, only the first two terms of the equation can be minimized. Hence, the fitting problem can be expressed as:

$$\min\left(\mathrm{NLF}\right)=\min\left(-\sum_{i=1}^{n}Q_{i}^{0}\;\ln\;Q(t_{i})+\sum_{i=1}^{n}Q(t_{i})\right),$$

subject to

$$\frac{d}{dt}X(t,P)=F(X,P,t),$$

Y(t,P)=Q(t,P),

$$X(0)=(S(0),S_{f}(0),I(0),Q(0),R(0)),$$

X(t,P)≥0.

However, this minimization problem provides the desired fitting with feasible parameter values if ℙ is identifiable, which was confirmed in two steps. First, the structural identifiability was examined, and next, the practical identifiability was confirmed.

Structural identifiability refers to the existence of a unique solution to *X*(*t*, ℙ) for each ℙ under initial conditions. If any component of ℙ is implicitly related, different values of ℙ may yield the same solution *X*(*t*, ℙ) for a given initial condition, which may hinder the unique estimation of parameter ℙ from the data.

The Fisher information matrix (FIM) is used to confirm the structural identifiability. For a set of observations at *n* distinct points, a system of 5-dimensional state vector, and 4-dimensional vector of parameters ℙ = (*p*_1_, *p*_2_, *p*_3_, *p*_4_) = (*β*, *β*_*f*_, *μ*_*f*_, *I*(0)). Thus, the sensitivity matrix S, consists of *n* time-dependent 1×4 blocks, $A(t_{i})$.


$$S=\left[\begin{array}{c} A(t_{1})\\ A(t_{2})\\ \vdots \\ A(t_{n}) \end{array}\right]$$

Here,

$$A(t_{i})=\left[\frac{\partial Q(t_{i},P)}{\partial p_{k}}\right]=\left[\begin{array}{ccc} \frac{\partial Q(t_{i},P)}{\partial p_{1}} & \cdots & \frac{\partial Q(t_{i},P)}{\partial p_{4}} \end{array}\right],\;i=1,\cdots ,n.$$

Here, S is called the sensitivity information matrix (see [36] for more details) and the 4×4 FIM is $M=S^{T}S$.

According to the aforementioned definition, the FIM for our problem consists of four columns corresponding to the parameters to be estimated. S is evaluated at ℙ^0^ and the small perturbation about $P^{0}=\pm 0.01\;\tilde{P}$ is denoted by $\Delta \tilde{P}$. Here $\tilde{P}$ denotes the estimated parameter values, that is, $\Delta \tilde{P}=\tilde{P}-P^{0}=\tilde{P}\mp 0.01\tilde{P}$. This local perturbation gives rise to a small perturbation, $\Delta X=X(t,\tilde{P})-X(t,P^{0})$. Next, the chain rule of differentiation is applied to obtain $\Delta X=S\Delta \tilde{P}$, indicating that *X*(*t*, ℙ) is structurally identifiable if $\Delta X=S\Delta \tilde{P}$ has a unique solution for $\Delta \tilde{P}$. This is only possible when the FIM rank is equal to the number of unknown parameters [36]. Because the rank of the FIM in all three waves is four, which is equal to the number of unknown parameters, the structural identifiability of the parameters in all three waves is ensured.

Practical identifiability, or ability to be estimated, refers to the sufficiency of available observations, as too few observations may be insufficient for fitting. To evaluate practical identifiability, the profile likelihood of parameters *β*, *β*_*f*_, and *μ*_*f*_ is computed along with the initial value *I*(0) for three different waves. Profile likelihood revealed the dependency of the NLF on individual parameters, which in turn helped determine the finite confidence intervals for each parameter; otherwise, practical non-identifiability is proved. The related profile likelihoods can be defined as

$$PL_{p_{i}}(p_{i})=\underset{p_{j\neq i}}{\min}\;\mathrm{NLF}(P),\;where\;p_{i}\in [\tilde{p_{i}}(1-0.01),\tilde{p_{i}}(1+0.01)].$$

To determine the confidence interval, we consider $2\left(\mathrm{NLF}(P)-\mathrm{NLF}(\hat{P})\right)\approx \chi_{4}^{2}$. Here, $\chi_{4}^{2}$ is representing the 95^th^ percentile range of the chi-square distribution with a degree of freedom equal to the number of unknown parameters. Therefore, the NLF threshold for the 95% confidence interval is $\mathrm{NLF}(\hat{P})+9.4877/2$.

**Fig 2:**
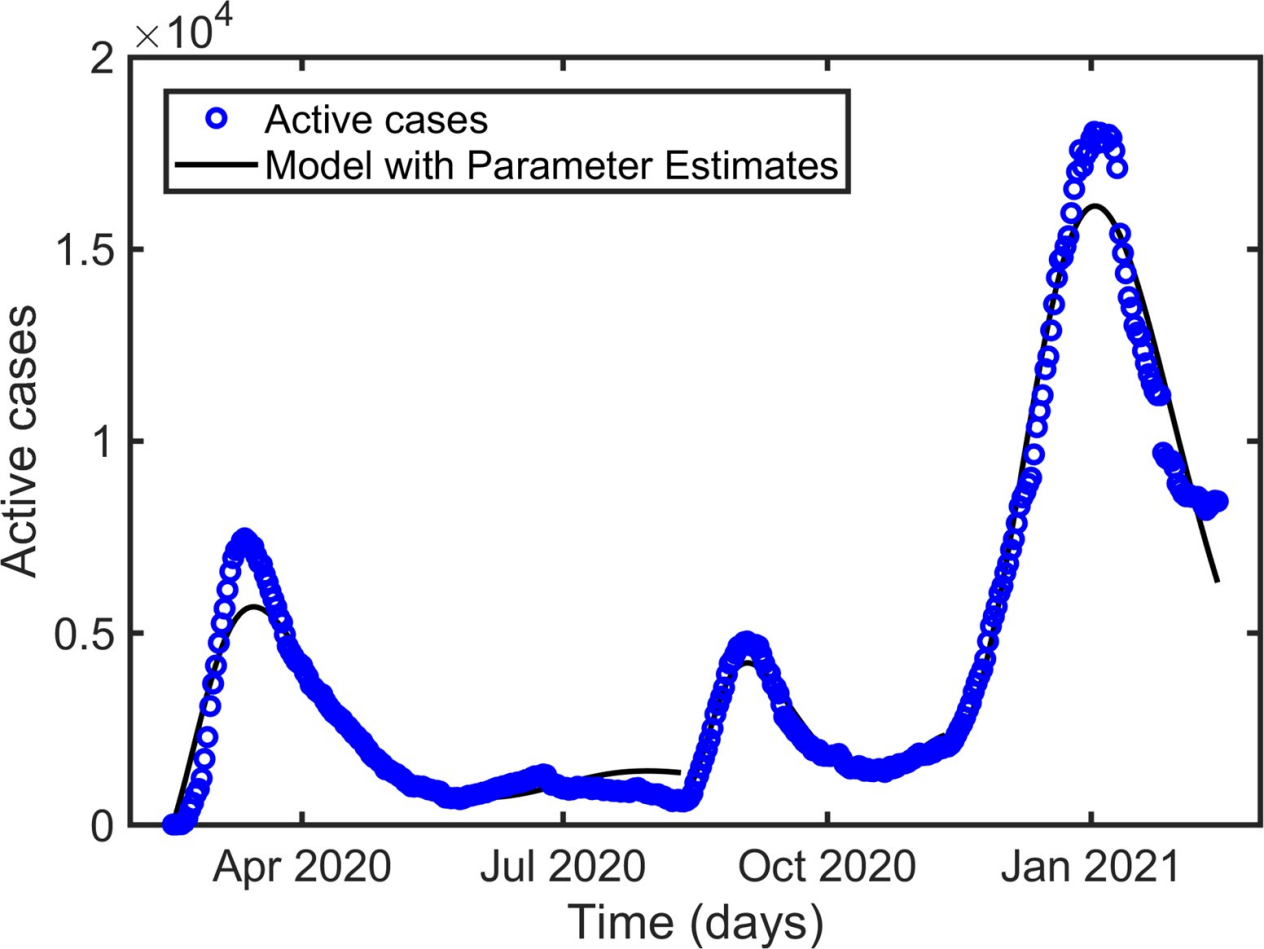
Curve fitting for the three waves of the active cases in South Korea.

**Fig 3:**
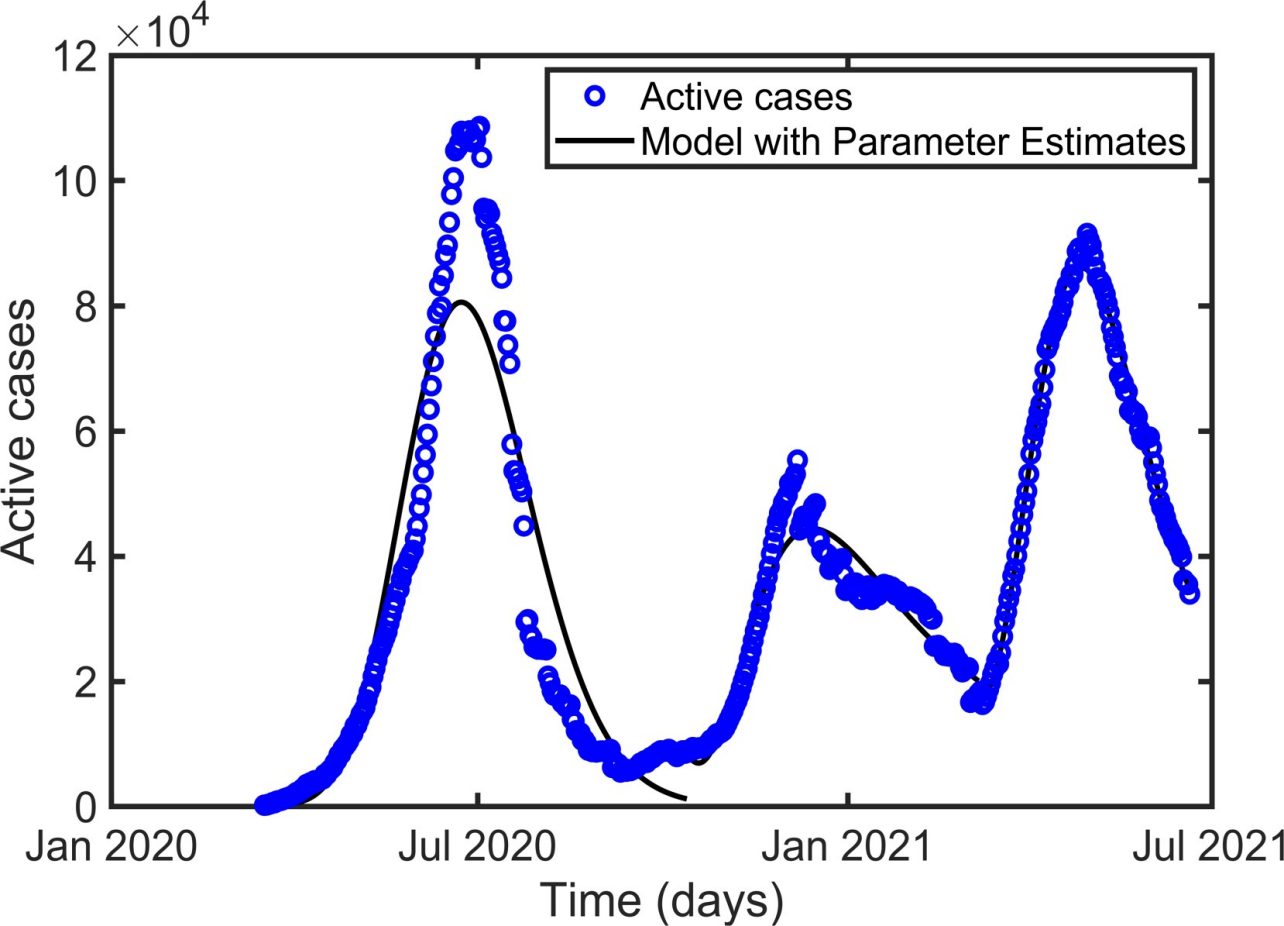
Curve fitting for the three waves of the active cases in Pakistan.

**Fig 4:**
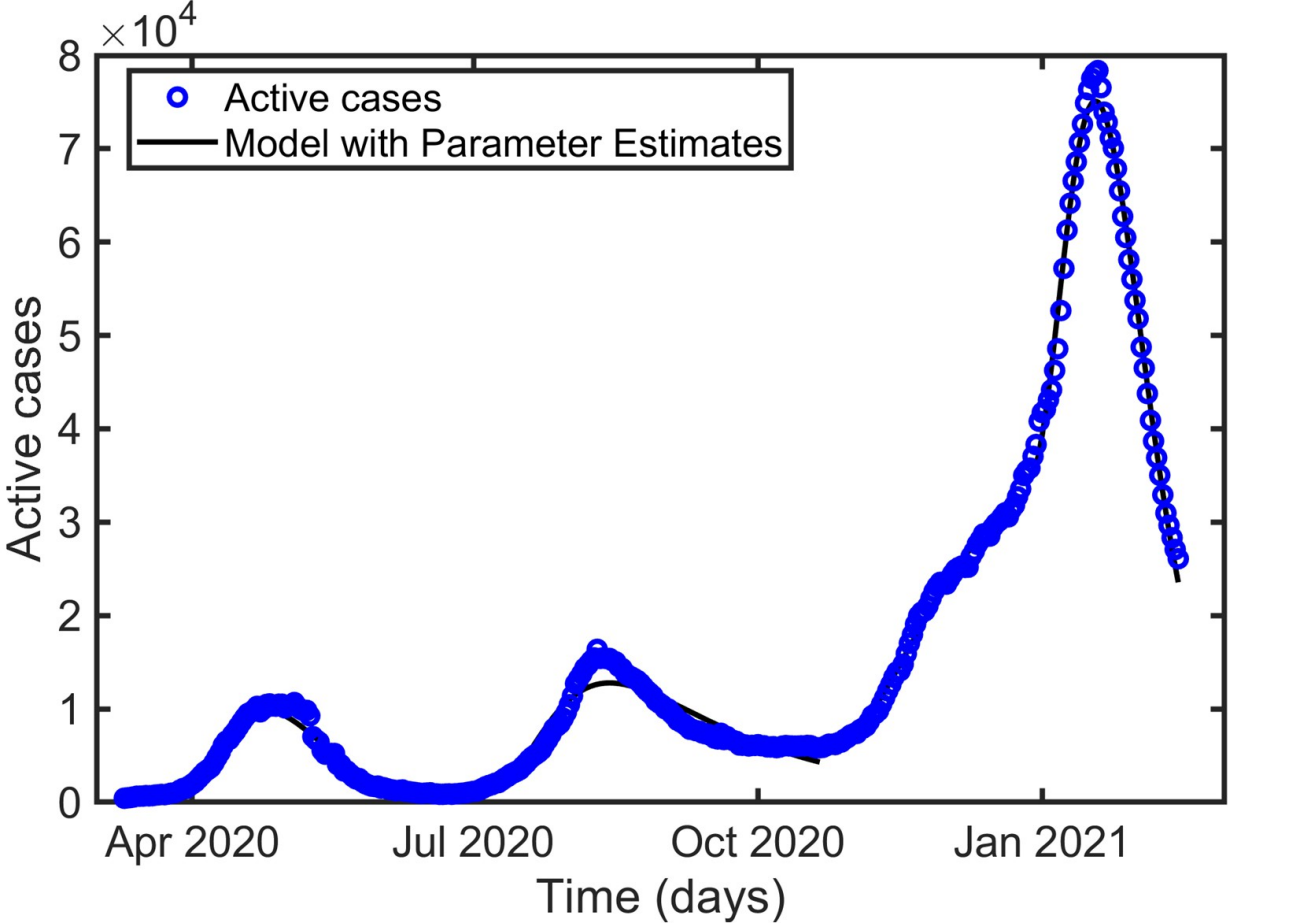
Curve fitting for the three waves of the active cases in Japan.

**Fig 5:**
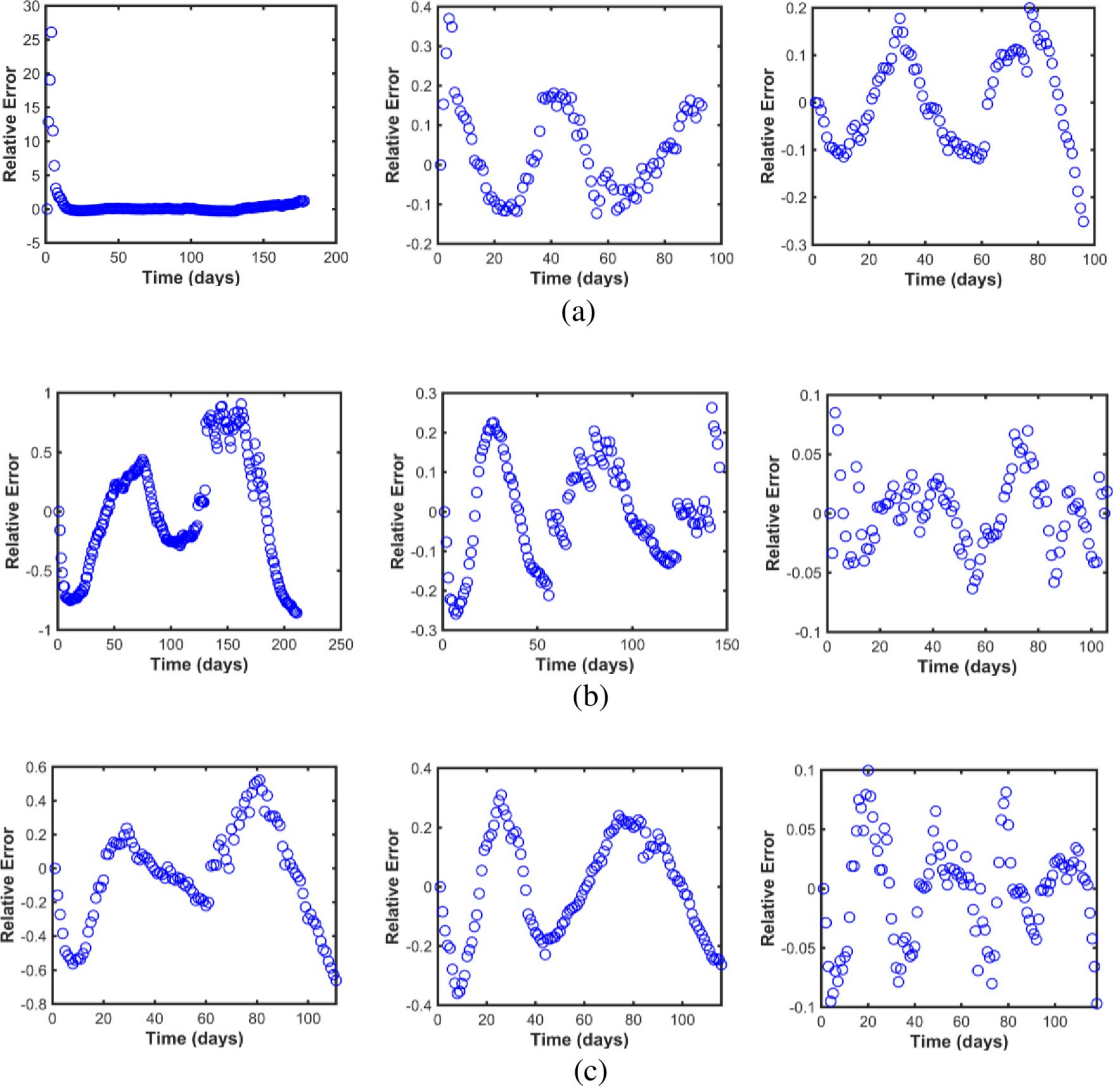
Relative error analysis graphs. (a) Data fitting relative errors for three waves in Korea. (b) Data fitting relative errors for three waves in Pakistan. (c) Data fitting relative errors for three waves in Japan.

Figs 2–4 represent the maximum likelihood fitting of the time series data for Korea, Pakistan, and Japan, respectively. While the graphs of relative error for Korea, Pakistan, and Japan are given in Fig 5. The estimated parameters are listed in Table 2, and the estimation of the infectious population, *I*(0), is presented in Tables 3–5 along with the 95% confidence intervals. In addition, the estimated parameter values are biologically meaningful.

**Table 3:** Initial values for the three different waves in the Republic of Korea. It is assumed that the initial value of the total population is *N*(0)≈52 million for all three waves [35]. Moreover, *S*_*f*_(0) = *N*(0)−*S*(0)−*I*(0)−*Q*(0)−*R*(0). Confidence intervals for *I*(0) in the first, second and third waves are (1001, 1047), (543, 590) and (708, 756), respectively.

Symbol	Initial Values			References
	1^st^ Wave Feb. 16 to Aug. 11	2^nd^ Wave Aug. 11 to Sep. 11	3^rd^ Wave Sep. 11 to Feb. 14	
*S*(*0*)	*N*(*0*)−*Q*(0)−*I*(0)−*R*(*0*)	34,271,000	18,691,000	Estimated
*I*(0)	1,024	566	732	Estimated
*Q*(*0*)	20	626	2,044	[33]
*R*(0)	9	13,729	25,266	[33]

**Table 4:** Initial values for the three different waves in Pakistan. It is assumed that the initial value of the total population is *N*(0)≈221 million for all three waves [35], where, *S*_*f*_(0) = *N*(0)−*S*(0)−*I*(0)−*Q*(0)−*R*(0). Confidence intervals for *I*(0) in the first, second and third waves are (53, 56), (756, 824) and (5097, 5271), respectively.

Symbol	Initial Values			References
	1^st^ Wave March 17 to Oct. 13	2^nd^ Wave Oct. 13 to March 7	3^rd^ Wave March 7 to June 20	
*S*(0)	*N*(*0*)−*Q*(0)−*I*(0)−*R*(*0*)	99,275,000	43,773,000	Estimated
*I*(0)	55	790	5,184	Estimated
*Q*(0)	234	8,651	18,055	[33]
*R*(0)	2	304,609	559,248	[33]

**Table 5:** Initial values for the three different waves in Japan. It is assumed that the initial value of the total population is *N*(0)≈125.8 million for all three waves [35], where, *S*_*f*_(0) = *N*(0)−*S*(0)−*I*(0)−*Q*(0)−*R*(0). Confidence intervals for *I*(0) in the first, second and third waves are (20, 25), (229, 247) and (1528, 1667), respectively.

Symbol	Initial Values			References
	1^st^ Wave March 10 to June 28	2^nd^ Wave June 28 to Oct. 21	3^rd^ Wave Oct. 21 to Feb 14	
*S*(0)	*N*(*0*)−*Q*(0)−*I*(0)−*R*(*0*)	55,487,000	33,291,000	Estimated
*I*(0)	23	238	1,598	Estimated
*Q*(0)	431	985	5840	[33]
*R*(0)	102	16452	87107	[33]

**Fig 6:**
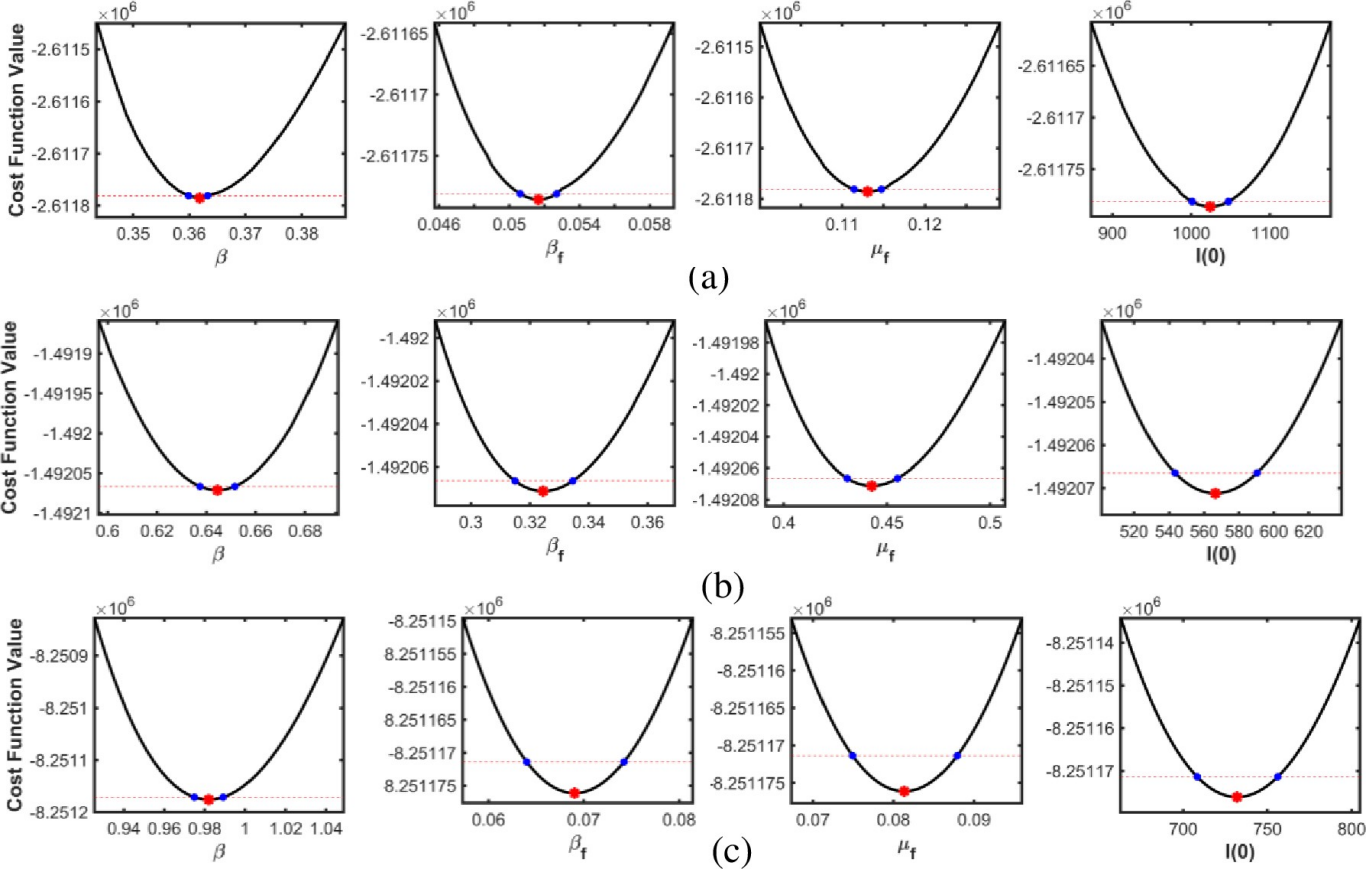
Estimated parameters versus the profile likelihood function for South Korea. (a), (b) and (c) show the graphs of estimated parameters versus profile likelihood function for the first, second, and third waves, respectively.

**Fig 7:**
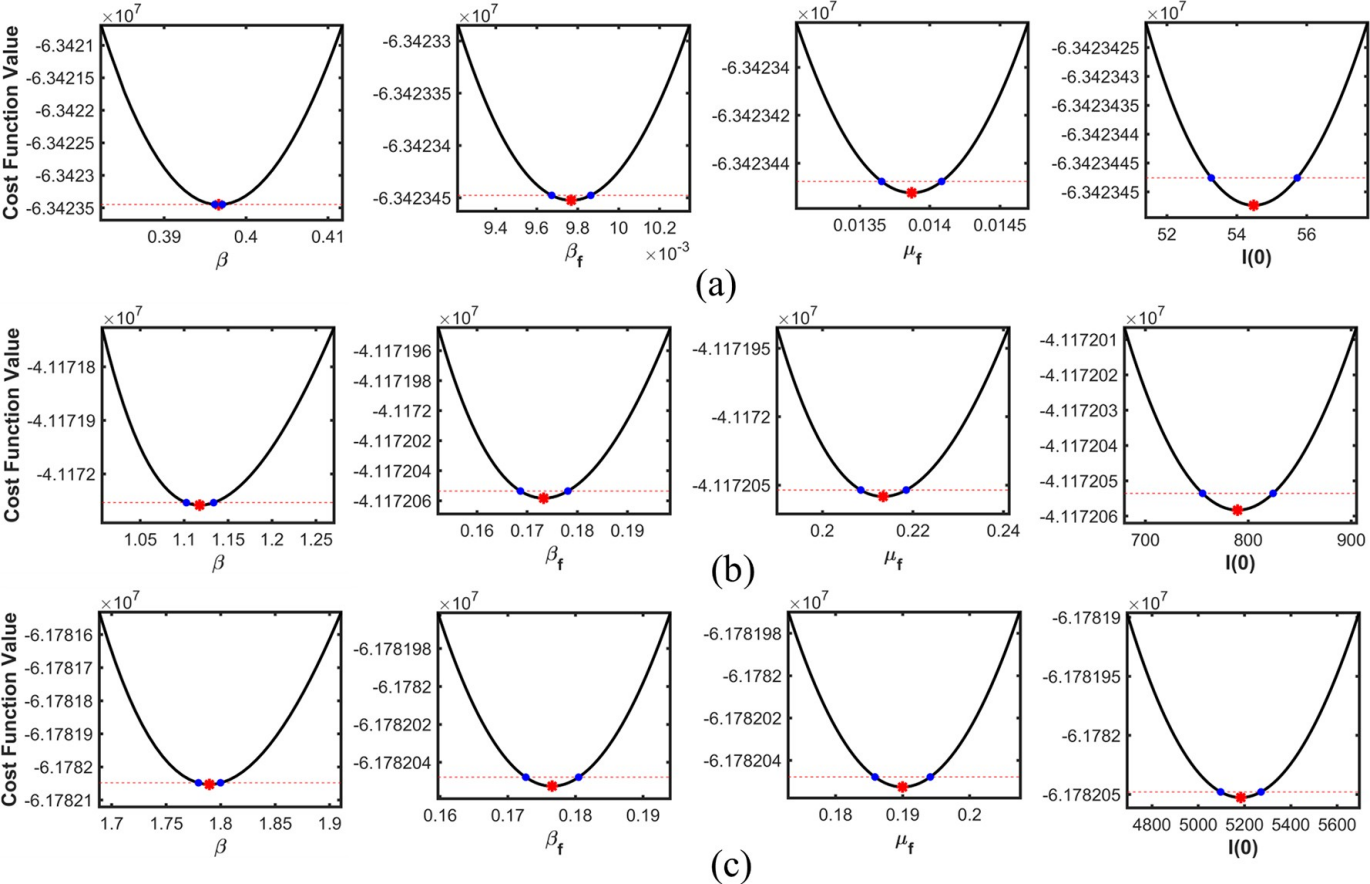
Estimated parameters versus the profile likelihood function for Pakistan. (a), (b), and (c) show the graphs of estimated parameters versus the profile likelihood function for the first, second, and third waves, respectively.

**Fig 8:**
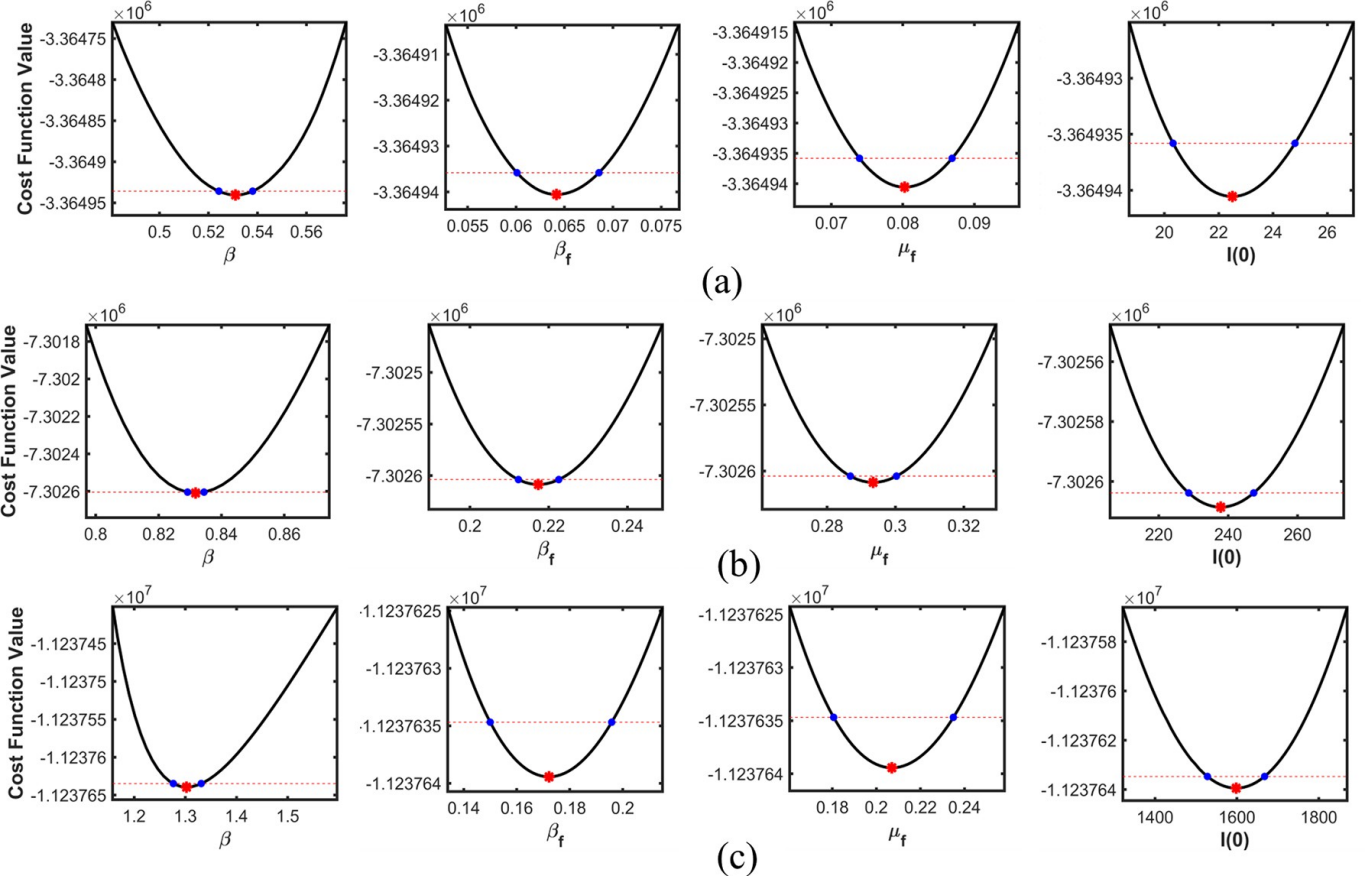
Estimated parameters versus the profile likelihood function for Japan. (a), (b), and (c) show the graphs of estimated parameters versus the profile likelihood function for the first, second, and third waves, respectively.

Figs 6–8 depict profile likelihoods, which reveal that NLF is minimized at the estimated parameter values, thus confirming practical identifiability.

## Results

**Fig 9:**
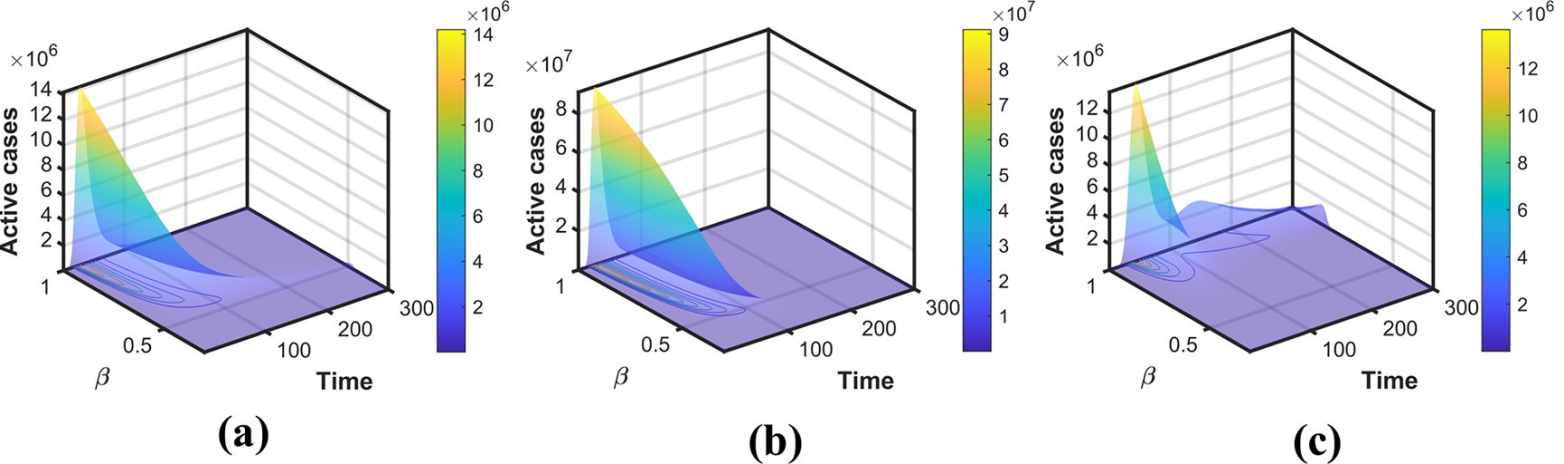
Simulation with different values of the transmission rate and time. The surface plots of the transmission rate, time, and active cases of South Korea, Pakistan, and Japan are presented in (a), (b), and (c), respectively.

A compartmental modeling approach is implemented with two different classes of susceptible individuals with different transmission rates. Individuals maintaining strict preventive measures were grouped as behavior-changed (aware) susceptible, *S*_*f*_. It is assumed that susceptible individuals from *S* compartment moved to *S*_*f*_ compartment upon realizing the high number of active cases or when the preventive measures (as a consequence of the high number of active cases) were strengthened by the public health authority at a rate *β*_*f*_. A behavior-changed individual moves from *S*_*f*_ to *S* following individuals in the *S* and *R* compartments at a rate *μ*_*f*_. By analyzing the dynamics of the model, it is confirmed that an outbreak occurs when the basic reproduction number *R*_0_ is above unity. It is worth noting that *R*_0_ increases with transmission rate *β* (Eq (8)). In addition, higher values of *β* are associated with a higher and quicker appearance of a peak of the active cases (please refer to Fig 9). The effect of the behavioral change on the intensity and timing of the peaks is discussed in the next subsection. Further, we have fitted our model to time-series data from South Korea, Pakistan, and Japan to understand the evolution of individual responses to public health guidelines.

### Behavioral response to public health guidelines modulates the peak

**Fig 10:**
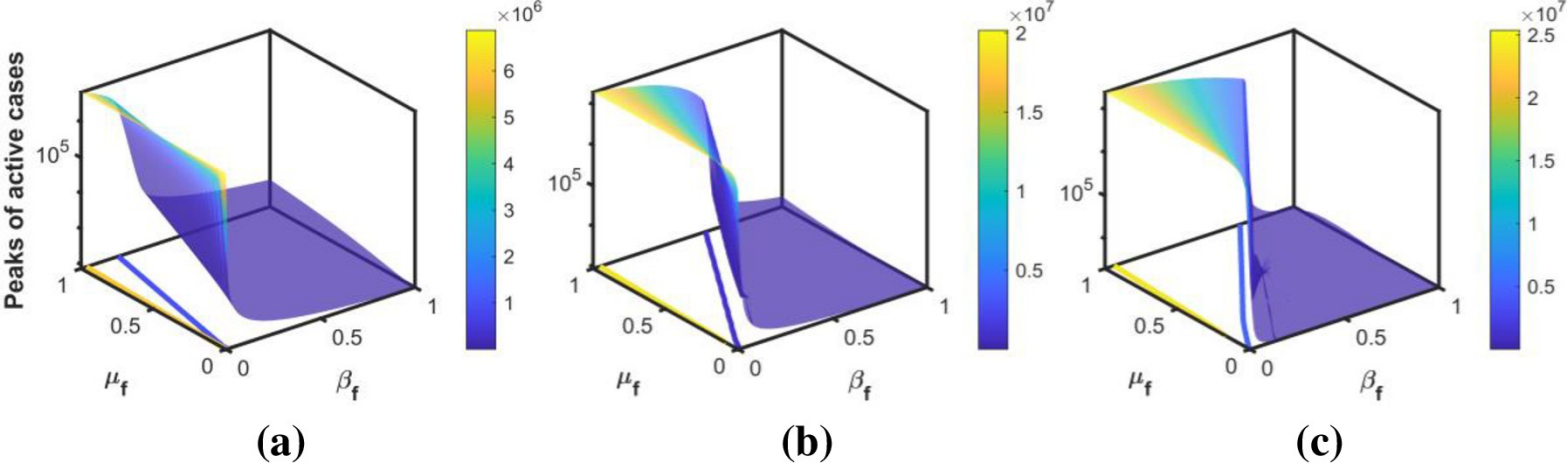
Sensitivity to the behavioral response. The surface plots show the sensitivity of the peak for active cases as a function of the behavior change rate and behavioral change ease rate during the (a) first, (b) second, and (c) third waves of South Korea, respectively.

**Fig 11:**
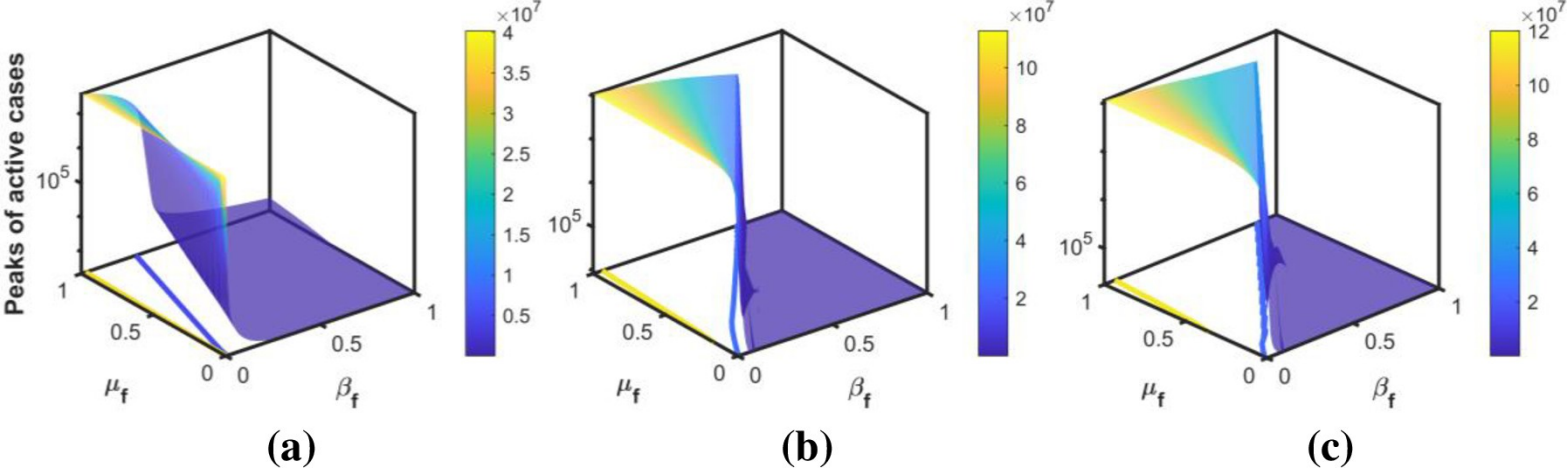
Sensitivity to the behavioral response. The surface plots show the sensitivity of the peak of active cases to behavior change rate and behavioral change ease rate during the (a) first, (b) second, and (c) third waves of Pakistan, respectively.

**Fig 12:**
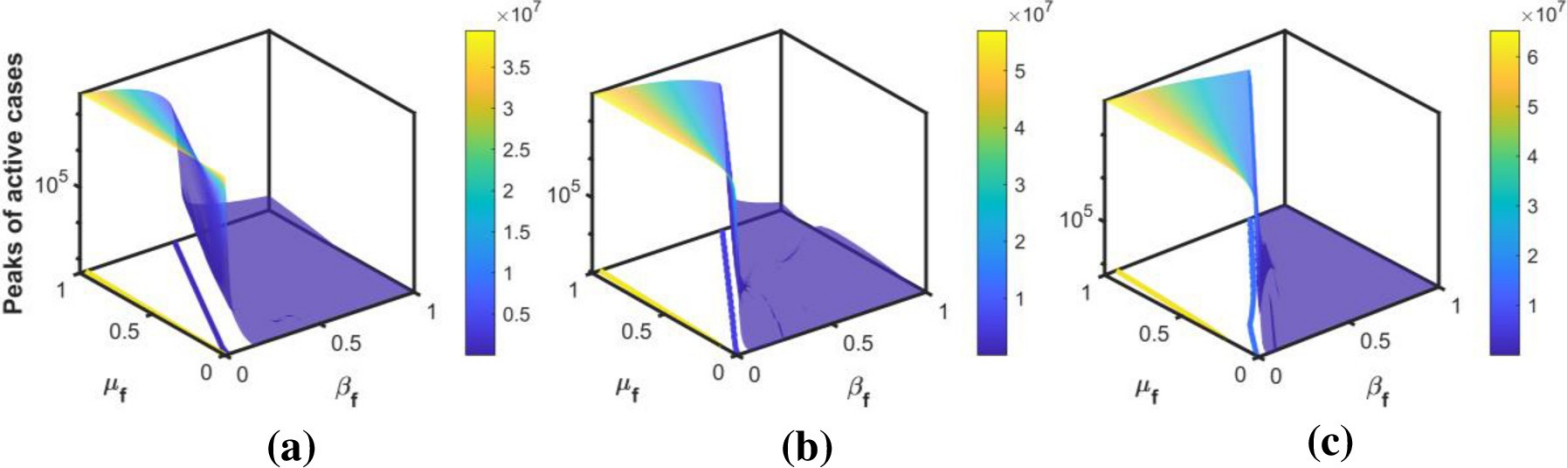
Sensitivity to the behavioral response. The surface plots show the sensitivity of the peak of active cases to behavior change rate and behavioral change ease rate during the (a) first, (b) second, and (c) third waves of Japan, respectively.

The intensity of the peak is modulated by the behavioral responses of individuals. Figs 10–12 show the peaks as a function of *β*_*f*_ and *μ*_*f*_ for the three peaks in Korea, Pakistan, and Japan, respectively. The surface plots show that for a fixed *β*_*f*_, the peak increases when people are negligent (i.e., *μ*_*f*_ increases) in following the public health guidelines. The intensity of the peak is more sensitive to *β*_*f*_ than to *μ*_*f*_. In addition, the sensitivity is higher for the set of values of *β*_*f*_ and *μ*_*f*_, lying between the blue and yellow contour lines in the *β*_*f*_−*μ*_*f*_ plane. For each value of *μ*_*f*_, if the value of *β*_*f*_ is above a threshold (shown by the blue contour line), the peak is minimized; that is, *maintaining a high behavior change rate is crucial for flattening the next outbreak*.

### Change in behavioral response during the pandemic: Case study of Korea, Pakistan, and Japan

According to [37], people indicated a minimum of one month and a maximum of two years (mean = 7.20 months) for a return to normal life. The proposed model was used for parameter estimations to examine the effect of individuals’ behavioral responses to prolonged public health restrictions established in response to the COVID-19 outbreak. According to our estimation the basic reproduction number *R*_0_, is 1.4474 (1.5861, 2.1237), 2.5777 (4.4701, 3.3268), and 3.9275 (7.1572, 5.2085) for the first, second, and third waves in Korea (Pakistan, Japan), respectively. Consistently increasing *R*_0_, in successive waves is a consequence of the appearance of highly transmissible mutants [38, 39]. Our estimates of *β* (Table 2) for the consecutive waves also followed the increasing transmissibility of the new strains. However, the intensity of the peaks did not follow the transmission rates (Figs 2–4). Each country has implemented different strategies to control the disease. Public health authorities implemented necessary actions such as screening, placing people in quarantine after confirmation, closing schools and offices, and making it compulsory to wear a mask in public places when the cases seemed to be high. As a result, in Korea, Pakistan, and Japan, *β*_*f*_ increased from the first to the second wave, indicating that more individuals became motivated to strictly follow public health guidelines, outweighing the effect of the increased transmission rate (*β*) and resulting in a smaller second wave than the first wave. It is worth noting that in Korea, Pakistan, and Japan, *μ*_*f*_ increased from the first to the second wave. As the intensity of the peak is more sensitive to *β*_*f*_ than to *μ*_*f*_ (as mentioned in the previous subsection), the effect of *β*_*f*_ dominates. Additionally, during the third peak, *β*_*f*_ decreased in Korea and Japan but remained constant in Pakistan (comparing columns 4 and 5 of Table 2). The third peak is the greatest in Korea and Japan because of poor awareness and strong transmissibility. Although the third peak in Pakistan is larger than the second in the country because of the increased transmissibility, constant awareness maintained it below the first peak.

For the estimated value of transmission rate in each wave, we plot the peaks of the active cases with respect to *β*_*f*_ and *μ*_*f*_ in Figs 10–12. These figures show that the peak of the active cases is lower when the values of *β*_*f*_ are high, indicating that when fear of the disease is high, the peak can be delayed and reduced. In the case of a higher behavioral change ease rate, the peaks are higher, and vice versa. This indicates that a reduction in the fear of the disease may cause more transmission in society and may cause the next wave. If the transmission rate is high, then, high fear of the disease would reduce the number of active cases.

## Discussion and conclusion

In this study, a mathematical model is used to explore the role of public acceptance of the public health guidelines during a pandemic outbreak. It is challenging to maintain a high level of awareness of preventive measures during a long-term disease with the emergence of subsequent outbreaks [37]. Therefore, a model that differentiates between susceptible and aware susceptible individuals in terms of disease transmission is proposed. Our analysis revealed that apart from the transmission rate and basic reproduction number, the behavior change rate modulates the intensity and timing of the peak. It is assumed that when individuals know their infection status, they are either hospitalized or isolated at home. Therefore, the infected class is not divided based on behavioral changes, as in the case of susceptible individuals. The fear of the disease causes susceptible individuals to move to the behavior-changed susceptible class, or because of monotonous maintenance of preventive measures, the behavior-changed susceptible individuals move back to the susceptible class. Our mathematical analysis confirmed that although *R*_0_ is the pivotal threshold for the COVID-19 outbreak, behavioral changes associated with preventive measures may have a considerable effect on the course of the pandemic.

Typically, data from active and recovered cases are used to fit the model, and the infectious compartment handles the spread of the disease. However, reliable data for the infectious class are not currently available because of a significant number of asymptomatic infectious individuals. To overcome this limitation, the initial values of the infectious class were estimated using the maximum-likelihood method. For a comprehensive analysis of the model, an identifiability analysis is performed to guarantee that the estimated unknown parameters were uniquely determined and that the numerical values of the estimated parameters were therefore meaningful.

Different techniques have been used to fit data with mathematical models, several of which have been described in [40–43]. In this study, the maximum likelihood method is used for fitting. As case studies, the COVID-19 outbreak in Korea, Pakistan, and Japan was investigated to determine how individual tendencies to follow preventive guidelines may affect the outbreak. The transmission, behavior change, and behavioral change ease rates were estimated for the three waves of the COVID-19 outbreak in South Korea, Pakistan, and Japan. The behavioral change rate reached an all-time high at the time of the second wave in Korea and Japan owing to government-imposed restrictions, such as school closures, public mask-wearing, and social isolation. However, it is not maintained in the third wave; therefore, the peak in this wave is the highest among the active cases.

Pakistan lacked diagnostic capabilities during the early stages of COVID-19; thus, suspicious samples were submitted to overseas labs [44]. Even though the transmission rate was the lowest during the first wave despite the low behavioral change rate and lack of testing facilities, the peak of this wave is the highest. Pakistan is then given test kits from China as well as primers from Japan [44]. Pakistan could examine samples from suspected cases nationwide. During the second wave, the fear level increased substantially. Compared to the second wave, the transmission rate of the third wave increased by 70%. However, the fear level is approximately the same as in the second wave. Therefore, Pakistan’s third peak is higher than its second.

It should be noted that a cross-country comparison is not carried out, as the country-based size of the peak and behavioral response would vary depending on the respective population size and socioeconomic conditions [45]. Rather, different peak scenarios were compared for each country. It is observed that in Korea Pakistan and Japan, individual responses to public health guidelines evolved with disease progression, and people resumed normal life, perceiving the risk as having declined. Usually, the fear of the disease prompts people to respond by limiting their contact; however, when they face insufficient incentives to alter their behavior, they return to normal behavior, which may modulate the size of the upcoming outbreak. Our mathematical findings highlight the critical necessity of behavioral change educational interventions, and community-level media campaigns. To address these issues, policymakers may improvise their strategy to make people more inclined to follow safety instructions [46].

## Supporting information

S1 File (DOCX)
