# Correction: Evolution and consequences of individual responses during the COVID-19 outbreak

**DOI:** 10.1371/journal.pone.0292775

**Published:** 2023-10-05

**Authors:** Wasim Abbas, Masud M. A., Anna Park, Sajida Parveen, Sangil Kim

There are errors in the Funding section. The correct Funding statement is: This work was supported by the National Research Foundation of Korea (NRF) Grant funded by the Korean Government (MSIP) and awarded to SK (NRF-2022R1A5A1033624) and AP (2022R1I1A1A01064105). The funders had no role in study design, data collection and analysis, decision to publish, or preparation of the manuscript.
