# Supplementary material for: Evolution and consequences of individual responses during the COVID-19 outbreak
Source: PLoS One. 2022 Sep 1;17(9):e0273964. doi: 10.1371/journal.pone.0273964 (PMC9436131; doi:10.1371/journal.pone.0273964)
Supplement: S1 File — (DOCX) [file pone.0273964.s001.docx]

**Data for active cases in South Korea and Pakistan**


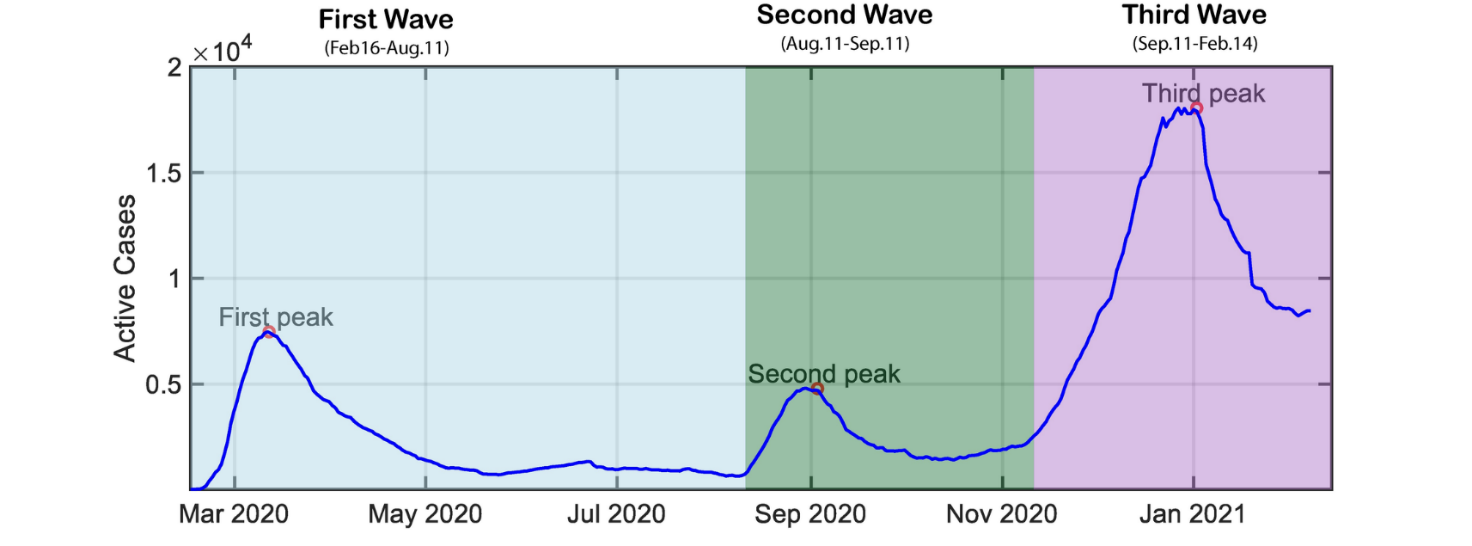


**S. Figure 1: Active cases in Korea^1^.**


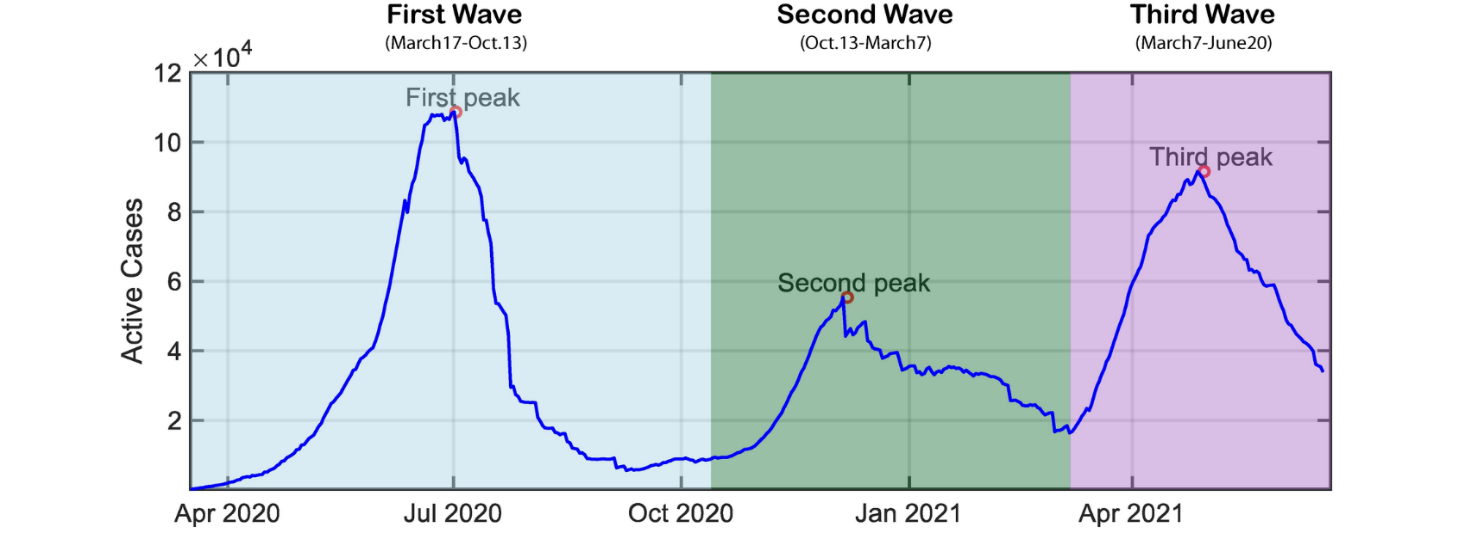


**S. Figure 2: Active cases in Pakistan^1^.**


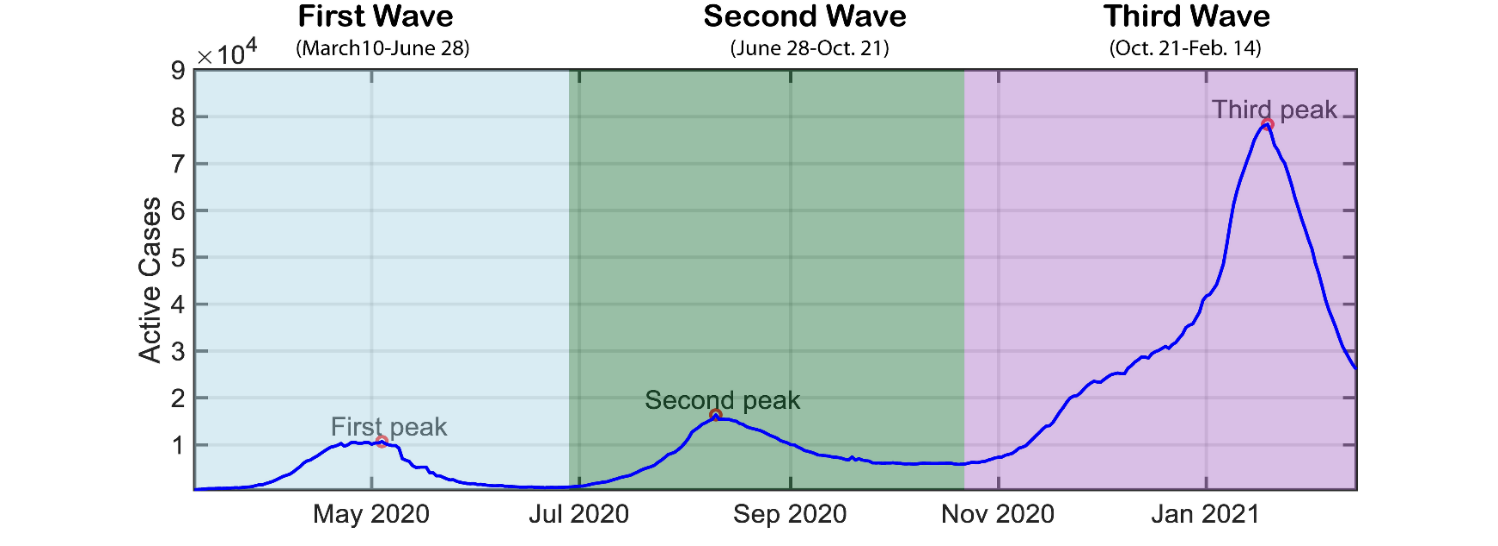


**S. Figure 3: Active cases in Japan**

**S. Table 1: Data of the active cases in South Korea^1^.**

| **Date** | **Active cases** | **Date** | **Active cases** | **Date** | **Active cases** |
| --- | --- | --- | --- | --- | --- |
| 2/16/2020 | 20 | 3/27/2020 | 4665 | 5/6/2020 | 1218 |
| 2/17/2020 | 20 | 3/28/2020 | 4523 | 5/7/2020 | 1135 |
| 2/18/2020 | 27 | 3/29/2020 | 4398 | 5/8/2020 | 1082 |
| 2/19/2020 | 30 | 3/30/2020 | 4275 | 5/9/2020 | 1016 |
| 2/20/2020 | 87 | 3/31/2020 | 4216 | 5/10/2020 | 1008 |
| 2/21/2020 | 186 | 4/1/2020 | 4155 | 5/11/2020 | 1021 |
| 2/22/2020 | 412 | 4/2/2020 | 3979 | 5/12/2020 | 1008 |
| 2/23/2020 | 584 | 4/3/2020 | 3867 | 5/13/2020 | 1008 |
| 2/24/2020 | 814 | 4/4/2020 | 3654 | 5/14/2020 | 969 |
| 2/25/2020 | 945 | 4/5/2020 | 3591 | 5/15/2020 | 937 |
| 2/26/2020 | 1225 | 4/6/2020 | 3500 | 5/16/2020 | 924 |
| 2/27/2020 | 1727 | 4/7/2020 | 3445 | 5/17/2020 | 900 |
| 2/28/2020 | 2297 | 4/8/2020 | 3408 | 5/18/2020 | 898 |
| 2/29/2020 | 3105 | 4/9/2020 | 3246 | 5/19/2020 | 877 |
| 3/1/2020 | 3688 | 4/10/2020 | 3125 | 5/20/2020 | 781 |
| 3/2/2020 | 4159 | 4/11/2020 | 3026 | 5/21/2020 | 723 |
| 3/3/2020 | 4750 | 4/12/2020 | 2930 | 5/22/2020 | 716 |
| 3/4/2020 | 5255 | 4/13/2020 | 2873 | 5/23/2020 | 705 |
| 3/5/2020 | 5643 | 4/14/2020 | 2808 | 5/24/2020 | 711 |
| 3/6/2020 | 6132 | 4/15/2020 | 2750 | 5/25/2020 | 713 |
| 3/7/2020 | 6605 | 4/16/2020 | 2627 | 5/26/2020 | 681 |
| 3/8/2020 | 6954 | 4/17/2020 | 2576 | 5/27/2020 | 701 |
| 3/9/2020 | 7165 | 4/18/2020 | 2484 | 5/28/2020 | 735 |
| 3/10/2020 | 7212 | 4/19/2020 | 2385 | 5/29/2020 | 770 |
| 3/11/2020 | 7407 | 4/20/2020 | 2324 | 5/30/2020 | 774 |
| 3/12/2020 | 7470 | 4/21/2020 | 2233 | 5/31/2020 | 793 |
| 3/13/2020 | 7402 | 4/22/2020 | 2179 | 6/1/2020 | 811 |
| 3/14/2020 | 7300 | 4/23/2020 | 2051 | 6/2/2020 | 823 |
| 3/15/2020 | 7253 | 4/24/2020 | 1967 | 6/3/2020 | 850 |
| 3/16/2020 | 7024 | 4/25/2020 | 1843 | 6/4/2020 | 857 |
| 3/17/2020 | 6838 | 4/26/2020 | 1769 | 6/5/2020 | 889 |
| 3/18/2020 | 6789 | 4/27/2020 | 1731 | 6/6/2020 | 915 |
| 3/19/2020 | 6527 | 4/28/2020 | 1654 | 6/7/2020 | 951 |
| 3/20/2020 | 6325 | 4/29/2020 | 1593 | 6/8/2020 | 978 |
| 3/21/2020 | 6085 | 4/30/2020 | 1459 | 6/9/2020 | 989 |
| 3/22/2020 | 5884 | 5/1/2020 | 1454 | 6/10/2020 | 1015 |
| 3/23/2020 | 5684 | 5/2/2020 | 1407 | 6/11/2020 | 1017 |
| 3/24/2020 | 5410 | 5/3/2020 | 1360 | 6/12/2020 | 1057 |
| 3/25/2020 | 5281 | 5/4/2020 | 1332 | 6/13/2020 | 1083 |
| 3/26/2020 | 4966 | 5/5/2020 | 1267 | 6/14/2020 | 1089 |
| 6/15/2020 | 1114 | 7/27/2020 | 971 | 9/7/2020 | 4663 |
| 6/16/2020 | 1117 | 7/28/2020 | 896 | 9/8/2020 | 4455 |
| 6/17/2020 | 1145 | 7/29/2020 | 882 | 9/9/2020 | 4221 |
| 6/18/2020 | 1177 | 7/30/2020 | 837 | 9/10/2020 | 4037 |
| 6/19/2020 | 1191 | 7/31/2020 | 821 | 9/11/2020 | 3953 |
| 6/20/2020 | 1237 | 8/1/2020 | 802 | 9/12/2020 | 3671 |
| 6/21/2020 | 1273 | 8/2/2020 | 806 | 9/13/2020 | 3592 |
| 6/22/2020 | 1277 | 8/3/2020 | 808 | 9/14/2020 | 3433 |
| 6/23/2020 | 1295 | 8/4/2020 | 770 | 9/15/2020 | 3146 |
| 6/24/2020 | 1324 | 8/5/2020 | 748 | 9/16/2020 | 2827 |
| 6/25/2020 | 1307 | 8/6/2020 | 696 | 9/17/2020 | 2742 |
| 6/26/2020 | 1148 | 8/7/2020 | 673 | 9/18/2020 | 2635 |
| 6/27/2020 | 1054 | 8/8/2020 | 629 | 9/19/2020 | 2545 |
| 6/28/2020 | 1069 | 8/9/2020 | 651 | 9/20/2020 | 2434 |
| 6/29/2020 | 1046 | 8/10/2020 | 663 | 9/21/2020 | 2412 |
| 6/30/2020 | 980 | 8/11/2020 | 626 | 9/22/2020 | 2277 |
| 7/1/2020 | 955 | 8/12/2020 | 623 | 9/23/2020 | 2178 |
| 7/2/2020 | 974 | 8/13/2020 | 648 | 9/24/2020 | 2116 |
| 7/3/2020 | 926 | 8/14/2020 | 705 | 9/25/2020 | 2082 |
| 7/4/2020 | 936 | 8/15/2020 | 833 | 9/26/2020 | 1951 |
| 7/5/2020 | 976 | 8/16/2020 | 1103 | 9/27/2020 | 1962 |
| 7/6/2020 | 1005 | 8/17/2020 | 1293 | 9/28/2020 | 1963 |
| 7/7/2020 | 982 | 8/18/2020 | 1521 | 9/29/2020 | 1822 |
| 7/8/2020 | 988 | 8/19/2020 | 1746 | 9/30/2020 | 1809 |
| 7/9/2020 | 987 | 8/20/2020 | 1976 | 10/1/2020 | 1808 |
| 7/10/2020 | 985 | 8/21/2020 | 2241 | 10/2/2020 | 1803 |
| 7/11/2020 | 941 | 8/22/2020 | 2524 | 10/3/2020 | 1820 |
| 7/12/2020 | 950 | 8/23/2020 | 2890 | 10/4/2020 | 1825 |
| 7/13/2020 | 986 | 8/24/2020 | 3137 | 10/5/2020 | 1856 |
| 7/14/2020 | 941 | 8/25/2020 | 3349 | 10/6/2020 | 1734 |
| 7/15/2020 | 914 | 8/26/2020 | 3585 | 10/7/2020 | 1594 |
| 7/16/2020 | 925 | 8/27/2020 | 3932 | 10/8/2020 | 1532 |
| 7/17/2020 | 919 | 8/28/2020 | 4210 | 10/9/2020 | 1479 |
| 7/18/2020 | 898 | 8/29/2020 | 4314 | 10/10/2020 | 1494 |
| 7/19/2020 | 894 | 8/30/2020 | 4473 | 10/11/2020 | 1481 |
| 7/20/2020 | 903 | 8/31/2020 | 4650 | 10/12/2020 | 1541 |
| 7/21/2020 | 877 | 9/1/2020 | 4660 | 10/13/2020 | 1508 |
| 7/22/2020 | 884 | 9/2/2020 | 4767 | 10/14/2020 | 1421 |
| 7/23/2020 | 883 | 9/3/2020 | 4786 | 10/15/2020 | 1467 |
| 7/24/2020 | 864 | 9/4/2020 | 4728 | 10/16/2020 | 1414 |
| 7/25/2020 | 928 | 9/5/2020 | 4668 | 10/17/2020 | 1407 |
| 7/26/2020 | 962 | 9/6/2020 | 4697 | 10/18/2020 | 1443 |
| 10/19/2020 | 1463 | 11/29/2020 | 5696 | 1/9/2021 | 17562 |
| 10/20/2020 | 1420 | 11/30/2020 | 6048 | 1/10/2021 | 17109 |
| 10/21/2020 | 1390 | 12/1/2020 | 6239 | 1/11/2021 | 15402 |
| 10/22/2020 | 1443 | 12/2/2020 | 6570 | 1/12/2021 | 14897 |
| 10/23/2020 | 1526 | 12/3/2020 | 6813 | 1/13/2021 | 14371 |
| 10/24/2020 | 1484 | 12/4/2020 | 7176 | 1/14/2021 | 13749 |
| 10/25/2020 | 1510 | 12/5/2020 | 7449 | 1/15/2021 | 13475 |
| 10/26/2020 | 1593 | 12/6/2020 | 7864 | 1/16/2021 | 13018 |
| 10/27/2020 | 1602 | 12/7/2020 | 8302 | 1/17/2021 | 12826 |
| 10/28/2020 | 1612 | 12/8/2020 | 8537 | 1/18/2021 | 12730 |
| 10/29/2020 | 1641 | 12/9/2020 | 8676 | 1/19/2021 | 12352 |
| 10/30/2020 | 1695 | 12/10/2020 | 8888 | 1/20/2021 | 12027 |
| 10/31/2020 | 1736 | 12/11/2020 | 9049 | 1/21/2021 | 11745 |
| 11/1/2020 | 1812 | 12/12/2020 | 9657 | 1/22/2021 | 11510 |
| 11/2/2020 | 1869 | 12/13/2020 | 10364 | 1/23/2021 | 11303 |
| 11/3/2020 | 1825 | 12/14/2020 | 10787 | 1/24/2021 | 11197 |
| 11/4/2020 | 1834 | 12/15/2020 | 11197 | 1/25/2021 | 11197 |
| 11/5/2020 | 1839 | 12/16/2020 | 11875 | 1/26/2021 | 9698 |
| 11/6/2020 | 1897 | 12/17/2020 | 12204 | 1/27/2021 | 9565 |
| 11/7/2020 | 1896 | 12/18/2020 | 12885 | 1/28/2021 | 9516 |
| 11/8/2020 | 1980 | 12/19/2020 | 13564 | 1/29/2021 | 9485 |
| 11/9/2020 | 2043 | 12/20/2020 | 14257 | 1/30/2021 | 9308 |
| 11/10/2020 | 2007 | 12/21/2020 | 14724 | 1/31/2021 | 8899 |
| 11/11/2020 | 2044 | 12/22/2020 | 14793 | 2/1/2021 | 8768 |
| 11/12/2020 | 2049 | 12/23/2020 | 15068 | 2/2/2021 | 8628 |
| 11/13/2020 | 2106 | 12/24/2020 | 15337 | 2/3/2021 | 8565 |
| 11/14/2020 | 2208 | 12/25/2020 | 15937 | 2/4/2021 | 8604 |
| 11/15/2020 | 2360 | 12/26/2020 | 16564 | 2/5/2021 | 8550 |
| 11/16/2020 | 2513 | 12/27/2020 | 17011 | 2/6/2021 | 8547 |
| 11/17/2020 | 2642 | 12/28/2020 | 17578 | 2/7/2021 | 8551 |
| 11/18/2020 | 2812 | 12/29/2020 | 17147 | 2/8/2021 | 8483 |
| 11/19/2020 | 3010 | 12/30/2020 | 17445 | 2/9/2021 | 8320 |
| 11/20/2020 | 3190 | 12/31/2020 | 17555 | 2/10/2021 | 8210 |
| 11/21/2020 | 3472 | 1/1/2021 | 17883 | 2/11/2021 | 8292 |
| 11/22/2020 | 3699 | 1/2/2021 | 18053 | 2/12/2021 | 8386 |
| 11/23/2020 | 3893 | 1/3/2021 | 17761 | 2/13/2021 | 8450 |
| 11/24/2020 | 4058 | 1/4/2021 | 18029 | 2/14/2021 | 8436 |
| 11/25/2020 | 4334 | 1/5/2021 | 17785 |  |  |
| 11/26/2020 | 4788 | 1/6/2021 | 17780 |  |  |
| 11/27/2020 | 5189 | 1/7/2021 | 17976 |  |  |
| 11/28/2020 | 5440 | 1/8/2021 | 17895 |  |  |

**S. Table 2: Active cases in Pakistan^1^.**

| **Date** | **Active Cases** | **Date** | **Active Cases** | **Date** | **Active Cases** |
| --- | --- | --- | --- | --- | --- |
| 3/17/2020 | 234 | 4/24/2020 | 8391 | 6/1/2020 | 44834 |
| 3/18/2020 | 270 | 4/25/2020 | 9216 | 6/2/2020 | 47667 |
| 3/19/2020 | 369 | 4/26/2020 | 9588 | 6/3/2020 | 49852 |
| 3/20/2020 | 462 | 4/27/2020 | 10018 | 6/4/2020 | 53366 |
| 3/21/2020 | 609 | 4/28/2020 | 10545 | 6/5/2020 | 56213 |
| 3/22/2020 | 629 | 4/29/2020 | 11529 | 6/6/2020 | 59467 |
| 3/23/2020 | 854 | 4/30/2020 | 11654 | 6/7/2020 | 63476 |
| 3/24/2020 | 938 | 5/1/2020 | 12890 | 6/8/2020 | 67249 |
| 3/25/2020 | 993 | 5/2/2020 | 12982 | 6/9/2020 | 71127 |
| 3/26/2020 | 1100 | 5/3/2020 | 13846 | 6/10/2020 | 75139 |
| 3/27/2020 | 1264 | 5/4/2020 | 14773 | 6/11/2020 | 78789 |
| 3/28/2020 | 1374 | 5/5/2020 | 15233 | 6/12/2020 | 83223 |
| 3/29/2020 | 1483 | 5/6/2020 | 15807 | 6/13/2020 | 79798 |
| 3/30/2020 | 1601 | 5/7/2020 | 17045 | 6/14/2020 | 84863 |
| 3/31/2020 | 1764 | 5/8/2020 | 18306 | 6/15/2020 | 88028 |
| 4/1/2020 | 1963 | 5/9/2020 | 19100 | 6/16/2020 | 89692 |
| 4/2/2020 | 2248 | 5/10/2020 | 20803 | 6/17/2020 | 93348 |
| 4/3/2020 | 2297 | 5/11/2020 | 22062 | 6/18/2020 | 97810 |
| 4/4/2020 | 2537 | 5/12/2020 | 23395 | 6/19/2020 | 100450 |
| 4/5/2020 | 2870 | 5/13/2020 | 24787 | 6/20/2020 | 104780 |
| 4/6/2020 | 2970 | 5/14/2020 | 25323 | 6/21/2020 | 105224 |
| 4/7/2020 | 3521 | 5/15/2020 | 26260 | 6/22/2020 | 106040 |
| 4/8/2020 | 3667 | 5/16/2020 | 27085 | 6/23/2020 | 107868 |
| 4/9/2020 | 3827 | 5/17/2020 | 27937 | 6/24/2020 | 107417 |
| 4/10/2020 | 3696 | 5/18/2020 | 29300 | 6/25/2020 | 107760 |
| 4/11/2020 | 4131 | 5/19/2020 | 30538 | 6/26/2020 | 107615 |
| 4/12/2020 | 4056 | 5/20/2020 | 31812 | 6/27/2020 | 107942 |
| 4/13/2020 | 4186 | 5/21/2020 | 32919 | 6/28/2020 | 106213 |
| 4/14/2020 | 4363 | 5/22/2020 | 34426 | 6/29/2020 | 106938 |
| 4/15/2020 | 4435 | 5/23/2020 | 34683 | 6/30/2020 | 106530 |
| 4/16/2020 | 5146 | 5/24/2020 | 36270 | 7/1/2020 | 108273 |
| 4/17/2020 | 5125 | 5/25/2020 | 37700 | 7/2/2020 | 108642 |
| 4/18/2020 | 5663 | 5/26/2020 | 38194 | 7/3/2020 | 103722 |
| 4/19/2020 | 5966 | 5/27/2020 | 38784 | 7/4/2020 | 95570 |
| 4/20/2020 | 6272 | 5/28/2020 | 39736 | 7/5/2020 | 93932 |
| 4/21/2020 | 6958 | 5/29/2020 | 40406 | 7/6/2020 | 95407 |
| 4/22/2020 | 7384 | 5/30/2020 | 40931 | 7/7/2020 | 94713 |
| 4/23/2020 | 8246 | 5/31/2020 | 42742 | 7/8/2020 | 91602 |
| 7/9/2020 | 90554 | 8/20/2020 | 11945 | 10/1/2020 | 8825 |
| 7/10/2020 | 89449 | 8/21/2020 | 11790 | 10/2/2020 | 8877 |
| 7/11/2020 | 88094 | 8/22/2020 | 10626 | 10/3/2020 | 8884 |
| 7/12/2020 | 86975 | 8/23/2020 | 10694 | 10/4/2020 | 9135 |
| 7/13/2020 | 84442 | 8/24/2020 | 10188 | 10/5/2020 | 8907 |
| 7/14/2020 | 77628 | 8/25/2020 | 9031 | 10/6/2020 | 8588 |
| 7/15/2020 | 77573 | 8/26/2020 | 8987 | 10/7/2020 | 8528 |
| 7/16/2020 | 73751 | 8/27/2020 | 8803 | 10/8/2020 | 8015 |
| 7/17/2020 | 70787 | 8/28/2020 | 8833 | 10/9/2020 | 8335 |
| 7/18/2020 | 57885 | 8/29/2020 | 8748 | 10/10/2020 | 8646 |
| 7/19/2020 | 53652 | 8/30/2020 | 8801 | 10/11/2020 | 8904 |
| 7/20/2020 | 53555 | 8/31/2020 | 8873 | 10/12/2020 | 8552 |
| 7/21/2020 | 52427 | 9/1/2020 | 8881 | 10/13/2020 | 8651 |
| 7/22/2020 | 51283 | 9/2/2020 | 8813 | 10/14/2020 | 8782 |
| 7/23/2020 | 50307 | 9/3/2020 | 8761 | 10/15/2020 | 9209 |
| 7/24/2020 | 44854 | 9/4/2020 | 8909 | 10/16/2020 | 9421 |
| 7/25/2020 | 29504 | 9/5/2020 | 9132 | 10/17/2020 | 9174 |
| 7/26/2020 | 29857 | 9/6/2020 | 6269 | 10/18/2020 | 9296 |
| 7/27/2020 | 27421 | 9/7/2020 | 6542 | 10/19/2020 | 9384 |
| 7/28/2020 | 26924 | 9/8/2020 | 6726 | 10/20/2020 | 9384 |
| 7/29/2020 | 25513 | 9/9/2020 | 6794 | 10/21/2020 | 9378 |
| 7/30/2020 | 25347 | 9/10/2020 | 5540 | 10/22/2020 | 9642 |
| 7/31/2020 | 25177 | 9/11/2020 | 5795 | 10/23/2020 | 9855 |
| 8/1/2020 | 25177 | 9/12/2020 | 6046 | 10/24/2020 | 10235 |
| 8/2/2020 | 25145 | 9/13/2020 | 5673 | 10/25/2020 | 10668 |
| 8/3/2020 | 25172 | 9/14/2020 | 5831 | 10/26/2020 | 10788 |
| 8/4/2020 | 25065 | 9/15/2020 | 5774 | 10/27/2020 | 11190 |
| 8/5/2020 | 20836 | 9/16/2020 | 5936 | 10/28/2020 | 11627 |
| 8/6/2020 | 19770 | 9/17/2020 | 6066 | 10/29/2020 | 11695 |
| 8/7/2020 | 18494 | 9/18/2020 | 6295 | 10/30/2020 | 11864 |
| 8/8/2020 | 17815 | 9/19/2020 | 6572 | 10/31/2020 | 12121 |
| 8/9/2020 | 17791 | 9/20/2020 | 6952 | 11/1/2020 | 12592 |
| 8/10/2020 | 17799 | 9/21/2020 | 7015 | 11/2/2020 | 13242 |
| 8/11/2020 | 17833 | 9/22/2020 | 7303 | 11/3/2020 | 13965 |
| 8/12/2020 | 16599 | 9/23/2020 | 7070 | 11/4/2020 | 14646 |
| 8/13/2020 | 16475 | 9/24/2020 | 7388 | 11/5/2020 | 15317 |
| 8/14/2020 | 15932 | 9/25/2020 | 7831 | 11/6/2020 | 16242 |
| 8/15/2020 | 16261 | 9/26/2020 | 7797 | 11/7/2020 | 16912 |
| 8/16/2020 | 16248 | 9/27/2020 | 8205 | 11/8/2020 | 17804 |
| 8/17/2020 | 13953 | 9/28/2020 | 8353 | 11/9/2020 | 18981 |
| 8/18/2020 | 13633 | 9/29/2020 | 8702 | 11/10/2020 | 20045 |
| 8/19/2020 | 12116 | 9/30/2020 | 8903 | 11/11/2020 | 21098 |
| 11/12/2020 | 22088 | 12/24/2020 | 38268 | 2/4/2021 | 32889 |
| 11/13/2020 | 23641 | 12/25/2020 | 38511 | 2/5/2021 | 32454 |
| 11/14/2020 | 24938 | 12/26/2020 | 39177 | 2/6/2021 | 32514 |
| 11/15/2020 | 26538 | 12/27/2020 | 39329 | 2/7/2021 | 32265 |
| 11/16/2020 | 28048 | 12/28/2020 | 39488 | 2/8/2021 | 31983 |
| 11/17/2020 | 29055 | 12/29/2020 | 39599 | 2/9/2021 | 31510 |
| 11/18/2020 | 30362 | 12/30/2020 | 37080 | 2/10/2021 | 30512 |
| 11/19/2020 | 32005 | 12/31/2020 | 34537 | 2/11/2021 | 30225 |
| 11/20/2020 | 33839 | 1/1/2021 | 34773 | 2/12/2021 | 29981 |
| 11/21/2020 | 34974 | 1/2/2021 | 35130 | 2/13/2021 | 25649 |
| 11/22/2020 | 36683 | 1/3/2021 | 35663 | 2/14/2021 | 25635 |
| 11/23/2020 | 38348 | 1/4/2021 | 35722 | 2/15/2021 | 25747 |
| 11/24/2020 | 40379 | 1/5/2021 | 35707 | 2/16/2021 | 25383 |
| 11/25/2020 | 42115 | 1/6/2021 | 33740 | 2/17/2021 | 25008 |
| 11/26/2020 | 43963 | 1/7/2021 | 34049 | 2/18/2021 | 24176 |
| 11/27/2020 | 45533 | 1/8/2021 | 33124 | 2/19/2021 | 24139 |
| 11/28/2020 | 46861 | 1/9/2021 | 33474 | 2/20/2021 | 24081 |
| 11/29/2020 | 47390 | 1/10/2021 | 34803 | 2/21/2021 | 24466 |
| 11/30/2020 | 48576 | 1/11/2021 | 35246 | 2/22/2021 | 24226 |
| 12/1/2020 | 49105 | 1/12/2021 | 34007 | 2/23/2021 | 24483 |
| 12/2/2020 | 49780 | 1/13/2021 | 33102 | 2/24/2021 | 23665 |
| 12/3/2020 | 51654 | 1/14/2021 | 33869 | 2/25/2021 | 23281 |
| 12/4/2020 | 51507 | 1/15/2021 | 34169 | 2/26/2021 | 22285 |
| 12/5/2020 | 52359 | 1/16/2021 | 33763 | 2/27/2021 | 21554 |
| 12/6/2020 | 53126 | 1/17/2021 | 34701 | 2/28/2021 | 21836 |
| 12/7/2020 | 55354 | 1/18/2021 | 34986 | 3/1/2021 | 22098 |
| 12/8/2020 | 44218 | 1/19/2021 | 35485 | 3/2/2021 | 22184 |
| 12/9/2020 | 45324 | 1/20/2021 | 35163 | 3/3/2021 | 16678 |
| 12/10/2020 | 46376 | 1/21/2021 | 35293 | 3/4/2021 | 17117 |
| 12/11/2020 | 44582 | 1/22/2021 | 34916 | 3/5/2021 | 17117 |
| 12/12/2020 | 45124 | 1/23/2021 | 35063 | 3/6/2021 | 17352 |
| 12/13/2020 | 46629 | 1/24/2021 | 34628 | 3/7/2021 | 18055 |
| 12/14/2020 | 47236 | 1/25/2021 | 33820 | 3/8/2021 | 18415 |
| 12/15/2020 | 48008 | 1/26/2021 | 34412 | 3/9/2021 | 16349 |
| 12/16/2020 | 48369 | 1/27/2021 | 33820 | 3/10/2021 | 16699 |
| 12/17/2020 | 42851 | 1/28/2021 | 33295 | 3/11/2021 | 17627 |
| 12/18/2020 | 42478 | 1/29/2021 | 32726 | 3/12/2021 | 18703 |
| 12/19/2020 | 40922 | 1/30/2021 | 33439 | 3/13/2021 | 19764 |
| 12/20/2020 | 40553 | 1/31/2021 | 33182 | 3/14/2021 | 21121 |
| 12/21/2020 | 40491 | 2/1/2021 | 33493 | 3/15/2021 | 22038 |
| 12/22/2020 | 40261 | 2/2/2021 | 33365 | 3/16/2021 | 23355 |
| 12/23/2020 | 37905 | 2/3/2021 | 33184 | 3/17/2021 | 22792 |
| 3/18/2021 | 24592 | 4/19/2021 | 82276 | 5/21/2021 | 63229 |
| 3/19/2021 | 27188 | 4/20/2021 | 83298 | 5/22/2021 | 63436 |
| 3/20/2021 | 29576 | 4/21/2021 | 83162 | 5/23/2021 | 62620 |
| 3/21/2021 | 31107 | 4/22/2021 | 84935 | 5/24/2021 | 62917 |
| 3/22/2021 | 33070 | 4/23/2021 | 84976 | 5/25/2021 | 62295 |
| 3/23/2021 | 34535 | 4/24/2021 | 86529 | 5/26/2021 | 60268 |
| 3/24/2021 | 36849 | 4/25/2021 | 88698 | 5/27/2021 | 59018 |
| 3/25/2021 | 37985 | 4/26/2021 | 89219 | 5/28/2021 | 58611 |
| 3/26/2021 | 40120 | 4/27/2021 | 87794 | 5/29/2021 | 58857 |
| 3/27/2021 | 42384 | 4/28/2021 | 88207 | 5/30/2021 | 58878 |
| 3/28/2021 | 44447 | 4/29/2021 | 89838 | 5/31/2021 | 59033 |
| 3/29/2021 | 46663 | 4/30/2021 | 91547 | 6/1/2021 | 57336 |
| 3/30/2021 | 48566 | 5/1/2021 | 90553 | 6/2/2021 | 55052 |
| 3/31/2021 | 50397 | 5/2/2021 | 89661 | 6/3/2021 | 53099 |
| 4/1/2021 | 53127 | 5/3/2021 | 87953 | 6/4/2021 | 51478 |
| 4/2/2021 | 56347 | 5/4/2021 | 86151 | 6/5/2021 | 48937 |
| 4/3/2021 | 58500 | 5/5/2021 | 84480 | 6/6/2021 | 47764 |
| 4/4/2021 | 60072 | 5/6/2021 | 84172 | 6/7/2021 | 47376 |
| 4/5/2021 | 61450 | 5/7/2021 | 83699 | 6/8/2021 | 46190 |
| 4/6/2021 | 63102 | 5/8/2021 | 82731 | 6/9/2021 | 44987 |
| 4/7/2021 | 64373 | 5/9/2021 | 81830 | 6/10/2021 | 44236 |
| 4/8/2021 | 66994 | 5/10/2021 | 80375 | 6/11/2021 | 43618 |
| 4/9/2021 | 69811 | 5/11/2021 | 78959 | 6/12/2021 | 42717 |
| 4/10/2021 | 73078 | 5/12/2021 | 76536 | 6/13/2021 | 42290 |
| 4/11/2021 | 73875 | 5/13/2021 | 75052 | 6/14/2021 | 41726 |
| 4/12/2021 | 75266 | 5/14/2021 | 73398 | 6/15/2021 | 40929 |
| 4/13/2021 | 76034 | 5/15/2021 | 71804 | 6/16/2021 | 39905 |
| 4/14/2021 | 76757 | 5/16/2021 | 68819 | 6/17/2021 | 36215 |
| 4/15/2021 | 77294 | 5/17/2021 | 68223 | 6/18/2021 | 35809 |
| 4/16/2021 | 78425 | 5/18/2021 | 67665 | 6/19/2021 | 35491 |
| 4/17/2021 | 79108 | 5/19/2021 | 66377 | 6/20/2021 | 33972 |
| 4/18/2021 | 80559 | 5/20/2021 | 66282 |  |  |

**S. Table 3: Active cases in Japan^1^.**

| **Date** | **Active Cases** | **Date** | **Active Cases** | **Date** | **Active Cases** |
| --- | --- | --- | --- | --- | --- |
| 3/10/2020 | 431 | 4/19/2020 | 9500 | 5/29/2020 | 1728 |
| 3/11/2020 | 473 | 4/20/2020 | 9652 | 5/30/2020 | 1689 |
| 3/12/2020 | 509 | 4/21/2020 | 9904 | 5/31/2020 | 1729 |
| 3/13/2020 | 564 | 4/22/2020 | 10272 | 6/1/2020 | 1514 |
| 3/14/2020 | 644 | 4/23/2020 | 9690 | 6/2/2020 | 1549 |
| 3/15/2020 | 650 | 4/24/2020 | 9986 | 6/3/2020 | 1449 |
| 3/16/2020 | 663 | 4/25/2020 | 10472 | 6/4/2020 | 1355 |
| 3/17/2020 | 707 | 4/26/2020 | 10532 | 6/5/2020 | 1299 |
| 3/18/2020 | 690 | 4/27/2020 | 10314 | 6/6/2020 | 1232 |
| 3/19/2020 | 708 | 4/28/2020 | 10295 | 6/7/2020 | 1276 |
| 3/20/2020 | 766 | 4/29/2020 | 10497 | 6/8/2020 | 1314 |
| 3/21/2020 | 794 | 4/30/2020 | 10497 | 6/9/2020 | 1156 |
| 3/22/2020 | 835 | 5/1/2020 | 10108 | 6/10/2020 | 1135 |
| 3/23/2020 | 813 | 5/2/2020 | 10379 | 6/11/2020 | 1086 |
| 3/24/2020 | 866 | 5/3/2020 | 10379 | 6/12/2020 | 1043 |
| 3/25/2020 | 961 | 5/4/2020 | 10678 | 6/13/2020 | 991 |
| 3/26/2020 | 993 | 5/5/2020 | 10230 | 6/14/2020 | 947 |
| 3/27/2020 | 1092 | 5/6/2020 | 9982 | 6/15/2020 | 959 |
| 3/28/2020 | 1297 | 5/7/2020 | 9817 | 6/16/2020 | 986 |
| 3/29/2020 | 1465 | 5/8/2020 | 9840 | 6/17/2020 | 1010 |
| 3/30/2020 | 1482 | 5/9/2020 | 9263 | 6/18/2020 | 904 |
| 3/31/2020 | 1740 | 5/10/2020 | 7026 | 6/19/2020 | 892 |
| 4/1/2020 | 1956 | 5/11/2020 | 6704 | 6/20/2020 | 854 |
| 4/2/2020 | 2200 | 5/12/2020 | 6451 | 6/21/2020 | 850 |
| 4/3/2020 | 2539 | 5/13/2020 | 5539 | 6/22/2020 | 874 |
| 4/4/2020 | 2898 | 5/14/2020 | 5152 | 6/23/2020 | 893 |
| 4/5/2020 | 3249 | 5/15/2020 | 5202 | 6/24/2020 | 857 |
| 4/6/2020 | 3487 | 5/16/2020 | 5224 | 6/25/2020 | 898 |
| 4/7/2020 | 3739 | 5/17/2020 | 5224 | 6/26/2020 | 921 |
| 4/8/2020 | 4226 | 5/18/2020 | 4035 | 6/27/2020 | 954 |
| 4/9/2020 | 4805 | 5/19/2020 | 4035 | 6/28/2020 | 985 |
| 4/10/2020 | 5347 | 5/20/2020 | 3363 | 6/29/2020 | 1044 |
| 4/11/2020 | 6080 | 5/21/2020 | 3363 | 6/30/2020 | 1102 |
| 4/12/2020 | 6548 | 5/22/2020 | 3047 | 7/1/2020 | 1164 |
| 4/13/2020 | 6746 | 5/23/2020 | 2724 | 7/2/2020 | 1189 |
| 4/14/2020 | 7212 | 5/24/2020 | 2500 | 7/3/2020 | 1341 |
| 4/15/2020 | 7604 | 5/25/2020 | 2528 | 7/4/2020 | 1497 |
| 4/16/2020 | 8120 | 5/26/2020 | 2188 | 7/5/2020 | 1678 |
| 4/17/2020 | 8610 | 5/27/2020 | 1990 | 7/6/2020 | 1795 |
| 4/18/2020 | 9077 | 5/28/2020 | 1852 | 7/7/2020 | 1896 |
| 7/8/2020 | 2033 | 8/19/2020 | 13866 | 9/30/2020 | 6090 |
| 7/9/2020 | 2100 | 8/20/2020 | 13601 | 10/1/2020 | 6162 |
| 7/10/2020 | 2320 | 8/21/2020 | 13423 | 10/2/2020 | 6062 |
| 7/11/2020 | 2564 | 8/22/2020 | 13244 | 10/3/2020 | 6044 |
| 7/12/2020 | 2752 | 8/23/2020 | 13046 | 10/4/2020 | 5932 |
| 7/13/2020 | 3005 | 8/24/2020 | 12740 | 10/5/2020 | 5922 |
| 7/14/2020 | 3166 | 8/25/2020 | 12352 | 10/6/2020 | 5949 |
| 7/15/2020 | 3320 | 8/26/2020 | 11979 | 10/7/2020 | 5853 |
| 7/16/2020 | 3505 | 8/27/2020 | 11699 | 10/8/2020 | 5916 |
| 7/17/2020 | 3859 | 8/28/2020 | 11392 | 10/9/2020 | 6016 |
| 7/18/2020 | 4173 | 8/29/2020 | 10875 | 10/10/2020 | 6070 |
| 7/19/2020 | 4564 | 8/30/2020 | 10638 | 10/11/2020 | 6037 |
| 7/20/2020 | 4863 | 8/31/2020 | 10410 | 10/12/2020 | 6022 |
| 7/21/2020 | 5099 | 9/1/2020 | 10016 | 10/13/2020 | 6009 |
| 7/22/2020 | 5332 | 9/2/2020 | 9988 | 10/14/2020 | 6014 |
| 7/23/2020 | 5629 | 9/3/2020 | 9546 | 10/15/2020 | 6039 |
| 7/24/2020 | 6224 | 9/4/2020 | 9224 | 10/16/2020 | 6033 |
| 7/25/2020 | 6700 | 9/5/2020 | 8779 | 10/17/2020 | 6086 |
| 7/26/2020 | 7266 | 9/6/2020 | 8595 | 10/18/2020 | 6059 |
| 7/27/2020 | 7906 | 9/7/2020 | 8405 | 10/19/2020 | 5915 |
| 7/28/2020 | 8149 | 9/8/2020 | 8154 | 10/20/2020 | 5822 |
| 7/29/2020 | 8432 | 9/9/2020 | 7826 | 10/21/2020 | 5840 |
| 7/30/2020 | 8995 | 9/10/2020 | 7774 | 10/22/2020 | 5882 |
| 7/31/2020 | 9623 | 9/11/2020 | 7670 | 10/23/2020 | 6040 |
| 8/1/2020 | 10449 | 9/12/2020 | 7602 | 10/24/2020 | 6219 |
| 8/2/2020 | 11406 | 9/13/2020 | 7427 | 10/25/2020 | 6213 |
| 8/3/2020 | 12708 | 9/14/2020 | 7342 | 10/26/2020 | 6180 |
| 8/4/2020 | 13232 | 9/15/2020 | 7272 | 10/27/2020 | 6381 |
| 8/5/2020 | 13743 | 9/16/2020 | 7108 | 10/28/2020 | 6450 |
| 8/6/2020 | 14372 | 9/17/2020 | 6867 | 10/29/2020 | 6734 |
| 8/7/2020 | 14700 | 9/18/2020 | 6784 | 10/30/2020 | 6931 |
| 8/8/2020 | 15115 | 9/19/2020 | 7380 | 10/31/2020 | 7208 |
| 8/9/2020 | 15458 | 9/20/2020 | 6723 | 11/1/2020 | 7332 |
| 8/10/2020 | 16378 | 9/21/2020 | 7028 | 11/2/2020 | 7377 |
| 8/11/2020 | 15413 | 9/22/2020 | 6735 | 11/3/2020 | 7762 |
| 8/12/2020 | 15471 | 9/23/2020 | 6610 | 11/4/2020 | 7942 |
| 8/13/2020 | 15392 | 9/24/2020 | 6517 | 11/5/2020 | 8161 |
| 8/14/2020 | 15397 | 9/25/2020 | 6135 | 11/6/2020 | 8653 |
| 8/15/2020 | 15155 | 9/26/2020 | 6108 | 11/7/2020 | 9279 |
| 8/16/2020 | 15031 | 9/27/2020 | 6127 | 11/8/2020 | 9490 |
| 8/17/2020 | 14539 | 9/28/2020 | 6032 | 11/9/2020 | 9793 |
| 8/18/2020 | 14342 | 9/29/2020 | 6088 | 11/10/2020 | 10496 |
| 11/11/2020 | 11193 | 12/17/2020 | 29855 | 1/23/2021 | 71147 |
| 11/12/2020 | 11938 | 12/18/2020 | 30131 | 1/24/2021 | 70061 |
| 11/13/2020 | 12615 | 12/19/2020 | 30560 | 1/25/2021 | 67865 |
| 11/14/2020 | 13390 | 12/20/2020 | 30998 | 1/26/2021 | 65494 |
| 11/15/2020 | 13970 | 12/21/2020 | 30570 | 1/27/2021 | 62748 |
| 11/16/2020 | 14075 | 12/22/2020 | 31363 | 1/28/2021 | 60492 |
| 11/17/2020 | 14781 | 12/23/2020 | 31804 | 1/29/2021 | 58113 |
| 11/18/2020 | 15914 | 12/24/2020 | 32723 | 1/30/2021 | 56023 |
| 11/19/2020 | 17025 | 12/25/2020 | 33574 | 1/31/2021 | 53757 |
| 11/20/2020 | 17963 | 12/26/2020 | 35012 | 2/1/2021 | 51805 |
| 11/21/2020 | 19068 | 12/27/2020 | 35511 | 2/2/2021 | 48758 |
| 11/22/2020 | 19966 | 12/28/2020 | 35784 | 2/3/2021 | 46507 |
| 11/23/2020 | 20370 | 12/30/2020 | 38315 | 2/4/2021 | 43781 |
| 11/24/2020 | 20491 | 12/31/2020 | 40809 | 2/5/2021 | 40906 |
| 11/25/2020 | 21025 | 1/1/2021 | 41744 | 2/6/2021 | 38690 |
| 11/26/2020 | 21847 | 1/2/2021 | 42064 | 2/7/2021 | 36903 |
| 11/27/2020 | 22587 | 1/3/2021 | 43094 | 2/8/2021 | 35027 |
| 11/28/2020 | 23133 | 1/4/2021 | 44175 | 2/9/2021 | 32928 |
| 11/29/2020 | 23551 | 1/5/2021 | 46260 | 2/10/2021 | 30970 |
| 11/30/2020 | 23399 | 1/6/2021 | 48578 | 2/11/2021 | 29663 |
| 12/1/2020 | 23367 | 1/7/2021 | 52667 | 2/12/2021 | 28328 |
| 12/2/2020 | 23927 | 1/8/2021 | 57183 | 2/13/2021 | 27081 |
| 12/3/2020 | 24467 | 1/9/2021 | 61282 | 2/14/2021 | 26089 |
| 12/4/2020 | 24929 | 1/10/2021 | 64155 | 2/3/2021 | 46507 |
| 12/5/2020 | 25146 | 1/11/2021 | 66555 | 2/4/2021 | 43781 |
| 12/6/2020 | 25306 | 1/12/2021 | 68597 | 2/5/2021 | 40906 |
| 12/7/2020 | 25161 | 1/13/2021 | 70691 | 2/6/2021 | 38690 |
| 12/8/2020 | 25179 | 1/14/2021 | 72636 | 2/7/2021 | 36903 |
| 12/9/2020 | 26336 | 1/15/2021 | 74885 | 2/8/2021 | 35027 |
| 12/10/2020 | 26895 | 1/16/2021 | 76336 | 2/9/2021 | 32928 |
| 12/11/2020 | 27639 | 1/17/2021 | 77531 | 2/10/2021 | 30970 |
| 12/12/2020 | 28136 | 1/18/2021 | 78095 | 2/11/2021 | 29663 |
| 12/13/2020 | 28743 | 1/19/2021 | 78374 | 2/12/2021 | 28328 |
| 12/14/2020 | 28709 | 1/20/2021 | 76545 | 2/13/2021 | 27081 |
| 12/15/2020 | 28511 | 1/21/2021 | 73916 | 2/14/2021 | 26089 |
| 12/16/2020 | 29423 | 1/22/2021 | 72826 |  |  |
|  |  |  |  |  |  |

1. Active cases of COVID-19 in South Korea, Pakistan and Japan. 2021. <https://coronaboard.kr/en/> (accessed 2022-06-19).
